# Integrating Brain and Biomechanical Models—A New Paradigm for Understanding Neuro-muscular Control

**DOI:** 10.3389/fnins.2018.00039

**Published:** 2018-02-06

**Authors:** Sebastian S. James, Chris Papapavlou, Alexander Blenkinsop, Alexander J. Cope, Sean R. Anderson, Konstantinos Moustakas, Kevin N. Gurney

**Affiliations:** ^1^Adaptive Behaviour Research Group, Department of Psychology, The University of Sheffield, Sheffield, United Kingdom; ^2^Insigneo Institute for In-Silico Medicine, The University of Sheffield, Sheffield, United Kingdom; ^3^Department of Electrical and Computer Engineering, The University of Patras, Patras, Greece; ^4^Department of Computer Science, The University of Sheffield, Sheffield, United Kingdom; ^5^Department of Automatic Control Systems Engineering, The University of Sheffield, Sheffield, United Kingdom

**Keywords:** integrated brain biomechanics, neuromuscular, neuromechanics, oculomotor, saccade, basal-ganglia

## Abstract

To date, realistic models of how the central nervous system governs behavior have been restricted in scope to the brain, brainstem or spinal column, as if these existed as disembodied organs. Further, the model is often exercised in relation to an *in vivo* physiological experiment with input comprising an impulse, a periodic signal or constant activation, and output as a pattern of neural activity in one or more neural populations. Any link to behavior is inferred only indirectly via these activity patterns. We argue that to discover the principles of operation of neural systems, it is necessary to express their behavior in terms of physical movements of a realistic motor system, and to supply inputs that mimic sensory experience. To do this with confidence, we must connect our brain models to neuro-muscular models and provide relevant visual and proprioceptive feedback signals, thereby closing the loop of the simulation. This paper describes an effort to develop just such an integrated brain and biomechanical system using a number of pre-existing models. It describes a model of the saccadic oculomotor system incorporating a neuromuscular model of the eye and its six extraocular muscles. The position of the eye determines how illumination of a retinotopic input population projects information about the location of a saccade target into the system. A pre-existing saccadic burst generator model was incorporated into the system, which generated motoneuron activity patterns suitable for driving the biomechanical eye. The model was demonstrated to make accurate saccades to a target luminance under a set of environmental constraints. Challenges encountered in the development of this model showed the importance of this integrated modeling approach. Thus, we exposed shortcomings in individual model components which were only apparent when these were supplied with the more plausible inputs available in a closed loop design. Consequently we were able to suggest missing functionality which the system would require to reproduce more realistic behavior. The construction of such closed-loop animal models constitutes a new paradigm of *computational neurobehavior* and promises a more thoroughgoing approach to our understanding of the brain's function as a controller for movement and behavior.

## 1. Introduction

The field of computational neuroscience has provided many *systems models* of the brain (Arai et al., 1994; Gancarz and Grossberg, 1998; Hazy et al., 2007; Blenkinsop et al., 2017). We refer to these as *mechanistic computational models*, meaning models which consist of populations of neural elements, interconnected in a biologically plausible manner, which simulate the operation of the brain. Whilst they differ in scale and complexity, these models all seek to describe the fundamental mechanisms behind common animal behaviors such as locomotion, threat evasion, reaching or feeding. However, none of the models cited here actually reproduce these behaviors. In each case, the activity in a certain population of neurons is taken to be representative of a behavioral outcome. In some cases, it *is* reasonable to take the activity of an internal population within the brain model as being representative of the induced behavior. For example, a choice made in a *go/no-go* task could be determined from activity in a population within a basal ganglia model (Nambu et al., 1990; Kühn et al., 2004). The decision to *go* is selected by a reduction of activity in this population; maintenance of activity implies *no-go*. To validate the model, the error rates which it generates could be compared with experimentally determined error rates in primate subjects. We refer to this as an *output assumption model* because the output is assumed to signify behavior. (An *input assumption model* assumes that sensory input produces some particular form of neural activity in an input population of the model).

However, we may be interested in reproducing accurate simulated *trajectories*, in order to find out how degradation of parts of the model affect movement. In Parkinson's Disease, degradation of the dopamine neurons originating in the substantia nigra pars compacta (SNc) causes diskinesia (Galvan and Wichmann, 2008), as well as abnormal network activity in the basal ganglia (Brown et al., 2001; McCarthy et al., 2011). Sufferers of the disease would be expected to produce abnormal decision-making *and* movement trajectories in a reach-to-the-correct-target task such as the one described in James et al. (2017). A model which sought to explore in detail the effects of the SNc degradation both on the decision making *and* on the movement dynamics would need a physically accurate virtual arm, as well as physically realistic sensory input for the brain. This is no less than a complete model of those sections of the brain and body which act to fulfill the task. Such a modeling effort, if successful, would result in a virtual robot capable of expressing behavior *in response to sensory input from its environment*. This would represent a paradigm shift in the field of computational neuroscience worthy of the new name of *computational neurobehavior*.

In an attempt to build a model combining brain, realistic biomechanics *and* sensory feedback, we sought to extend our previous work modeling the oculomotor system by adding a virtual, biomechanical eye model able to make physically realistic movements. The rotational state of the eye would then determine how visual features in the virtual world were projected back into the brain model. The existing model (Cope et al., 2017) is already able to capture sensory input and convert it into a neural signal, assumed to specify the target of a *saccadic eye movement*; a fast movement of the eyes which directs the fovea to a region of interest in the field of view. The oculomotor system is an excellent candidate for modeling because its movements can be specified with only three degrees of freedom, making it one of the simplest neuro-muscular systems in the body. It is nevertheless behaviorally interesting, as saccadic eye movements reveal information about decision making at a subconscious level (Deubel and Schneider, 1996; Reppert et al., 2015; Marcos and Genovesio, 2016). The modeling of the oculomotor system is served by a large body of behavioral data describing saccades (Walker et al., 1997; Tipper et al., 2001; Casteau and Vitu, 2012), many anatomical studies of the neural substrates involved (Meredith and Ramoa, 1998; Isa, 2002; Isa and Hall, 2009) and electrophysiological data linking these together (Hepp and Henn, 1983; Dorris et al., 1997; McPeek et al., 2003; Vokoun et al., 2011). Furthermore, in the context of building *behaving* systems, a realistic mechanism for gathering visual information is a necessary part of any model for which the behavior requires visual attention and decision making. This is obvious from extrinsic considerations—a subject must look at a scene to make decisions or navigate within it. It also follows for *intrinsic* reasons. For example, Howard and Tipper (1997) showed that visual cues affect reach trajectories and the same group later demonstrated that reaching affects the saccadic system (Tipper et al., 2001) suggesting a close relationship between these neural systems. Building a behaving oculomotor system will therefore assist future computational neurobehavioral modeling efforts that involve reaching.

Many neural populations are involved in the coding of saccadic eye movements, only a very brief overview is given here; for a review, see Munoz (2002). One pathway takes information from the retina directly into the superficial layers of the superior colliculus in the brainstem (Sterling, 1971; Linden and Perry, 1983; Wu et al., 1994). Activity within the superior colliculus then excites neurons in the pons, medulla and rostral mid-brain (Sparks, 2002) and finally the motor neurons, which innervate the extraocular muscles (Fuchs and Luschei, 1970; Sparks, 2002). This direct pathway is responsible for the low latency saccades called express saccades (Schiller et al., 1987; Edelman and Keller, 1996). Information from the retina is also processed by visual cortex which feeds through to the frontal eye fields in which activity is related to reflexive and voluntary saccades (Schall and Thompson, 1999). Activity build-up in the frontal eye fields is transferred to the intermediate layers of the superior colliculus (Stanton et al., 1988b) and is also processed by the basal ganglia, which participates in the selection of the winning saccade end point (Stanton et al., 1988a; Hikosaka et al., 2000). Although both cortical and subcortical paths produce a saccade target signal in the superior colliculus, it is also possible for animals to make relatively normal saccades even after the colliculus has been ablated (Wurtz and Goldberg, 1972; Aizawa and Wurtz, 1998), though express saccades are lost with collicular lesions (Schiller et al., 1987). This makes the superior colliculus a perplexing structure, being both critically involved in saccade target specification (Sparks and Nelson, 1987) and saccade dynamic control (Waitzman et al., 1991; Goossens and van Opstal, 2012) and yet dispensible. The “backup pathway” likely incorporates the oculomotor vermis and fastigial oculomotor region of the cerebellum which are known to participate in the specification, dynamics and adaptation of saccadic eye movements (Takagi et al., 1998; Kleine, 2003).

There is a long history of modeling the oculomotor system. For a comprehensive review, see Girard and Berthoz (2005). Models of individual sub-systems have been proposed for brainstem (Robinson, 1975; Scudder, 1988; Gancarz and Grossberg, 1998), cerebellum (Dean et al., 1994; Dean, 1995; Quaia et al., 1999) and superior colliculus (Arai et al., 1994; Massone, 1994; Marino et al., 2012; Morén et al., 2013). More recently, combined models have also been developed incorporating sensory input (Cope et al., 2017) and driving a second order differential equation representing the eye (Tabareau et al., 2007; N'Guyen et al., 2014; Thurat et al., 2015). None of these models has yet fully closed the loop to produce a behaving system operating freely within its environment. We argue that developing integrated, closed-loop models of behaving systems offers insights into the operation of neural systems that are not available from input- or output-assumption models.

## 2. Materials and methods

The integrated brain and biomechanical model described here is a development of the model in Cope et al. (2017), referred to here as the *Cope-Chambers model*. This was a rate-coded neural network model incorporating retinal populations, frontal eye fields (FEF), the basal ganglia (BG), and the superior colliculus (SC). The Cope-Chambers model takes as *input* the positions of luminances (of fixed shape and intensity) on a topographic map. While certain assumptions were made about the input—that a luminant input excites activity on a retinotopic layer, with computer code carrying out the transformation achieved in the brain by a neural connectivity map (Thivierge and Marcus, 2007)—it is nonetheless *not* an input-assumption model according to our definition because the activity generated in the neural input layer is modeled as a response to the luminances, rather than being crafted. In the Cope-Chambers model, the centroid of the activity in the deep layers of superior colliculus was assumed to accurately encode the location of the eye at the end of the saccade (Robinson, 1972; Wurtz and Goldberg, 1972; McIlwain, 1982; Van Gisbergen et al., 1987). This location was used to recalculate the positions of the luminances in the eye's frame of reference at each time step. Because a pattern of neural activity in the output population was assumed to have a behavioral outcome, it was thus an *output-assumption model*. The model included no brainstem populations other than superior colliculus, nor a neuromuscular model.

To the Cope-Chambers model, we added a brainstem model and a biomechanical eye model. The rate-coded brainstem model was taken from the literature (Gancarz and Grossberg, 1998) as the best-of-breed saccadic burst generator (Girard and Berthoz, 2005). The biomechanical eye was implemented using the biomechanical modeling framework OpenSim; the brain and brainstem were modeled using the SpineML toolchain. These will be described below, along with a review of the Cope-Chambers model, but first we will give a description of the co-ordinate systems that were used.

### 2.1. Co-ordinates in the world

Before describing the biomechanical eye and the brain model, which consisted of retinotopically mapped neural sheets, we describe the co-ordinate system used in the world. The eye was located at the origin of a three-dimensional, right-handed Cartesian co-ordinate system, with its fovea directed in the −*z* direction. There was a notional spherical screen which was also centered at the origin of the co-ordinate system and had a radius of 50 (in arbitrary units). The *fixation point* was the point on the screen at which the eye was initially directed. Onto the screen were projected target luminances, each of which having a position described by two co-ordinates; $\theta_{x}^{t}$, a rotation of the horizon plane about the *x* axis, and $\theta_{y}^{t}$, a rotation of the meridian plane about the *y* axis. The position is the intersection of these rotated planes with the spherical screen (disregarding the intersection point of these three surfaces behind the eye). Note that a luminance with positive $\theta_{x}^{t}$ was above the horizon of this world; one whose $\theta_{y}^{t}$ was positive lay to the left of the world's meridian. For this reason, many of the figures in this paper are plotted with −θ_*y*_ on the *x*-axis and θ_*x*_ on the *y*-axis so that targets that lay up and to the right in the world do so in the graphs, also.

Luminances were crosses of height and width subtending ±3° and whose “bars” were 2° thick. Luminances were oriented like + symbols with their vertical bar aligned with the meridian plane and their horizontal bar aligned with the horizon.

The eye's frame of reference was initially aligned with the world's frame of reference. At each timestep, the eye's rotational state (described by the Euler rotations θ_*x*_, θ_*y*_, θ_*z*_) was used to translate the three dimensional Cartesian co-ordinates of the luminances in the world frame into co-ordinates in the eye frame. The luminance co-ordinates in the eye's frame of reference were used to determine the input to the brain model.

### 2.2. Existing brain model

The brain model, excluding the brainstem, is a re-implementation of the Cope-Chambers model of reflexive saccadic behavior (Cope et al., 2017). Reflexive saccades are fast eye movements elicited by abrupt changes in the peripheral visual scene (reflexive saccades can occur also as a result of auditory and somatosensory stimuli, but these modalities are ignored in this model). A reflexive saccade has a starting position defined by the initial orientation of the eye and an end-point position in which the eye is directed toward a new target. Regardless of the number of targets within the visual scene, the brain must choose one location as the end-point, because the eyes can look only in one direction at a time. The functionality reproduced by the Cope-Chambers model is “the selection of the best target end-point for a reflexive saccade.” A competition such as this between incompatible movements is often referred to as an *action selection* problem (Norman and Shallice, 1986; Maes, 1989; Redgrave et al., 1999). The Cope-Chambers model is therefore a model of action-selection in the oculomotor system for reflexive saccades. One hypothesis for the rôle played by the basal ganglia (BG) is that the system performs *action selection* (Mink, 1996; Redgrave et al., 1999; Hikosaka et al., 2000). The Cope-Chambers model places the BG at the center of the oculomotor system; this follows the known anatomy of the region (Hikosaka et al., 2000) and provides a mechanism for action selection of the best saccade. The BG receives input indirectly from the superior colliculus, which has a retinotopic arrangement (Ottes et al., 1986).

The BG receives excitatory inputs directly from retinotopic regions of the cortex including the frontal eye fields (FEF), supplementary eye fields (SEF), lateral intraparietal cortex (LIP) and dorsolateral prefrontal cortex. The dorsolateral prefrontal cortex, which participates in voluntary saccades (Funahashi et al., 1993; Munoz and Everling, 2004), is not modeled because the model concerns reflexive rather than voluntary eye movements. Several other regions of the brain that are associated with eye movements are also omitted from the model. The early visual processing stream in cortex, from V1, through to the LIP is subsumed into a “sustained retinal” signal which arrives at FEF. The justification here is that the model reacts to simple luminant targets and does not need to carry out the feature extraction performed by these visual areas. The supplementary eye fields are involved in the programming of saccade sequences (Tehovnik et al., 2000) and memory guided saccades (Chen and Wise, 1995; Schlag, 2002). Lesions of SEF do not affect visually guided saccades (Gaymard et al., 1998) and so the SEF is also omitted from the model.

**Figure 1:**
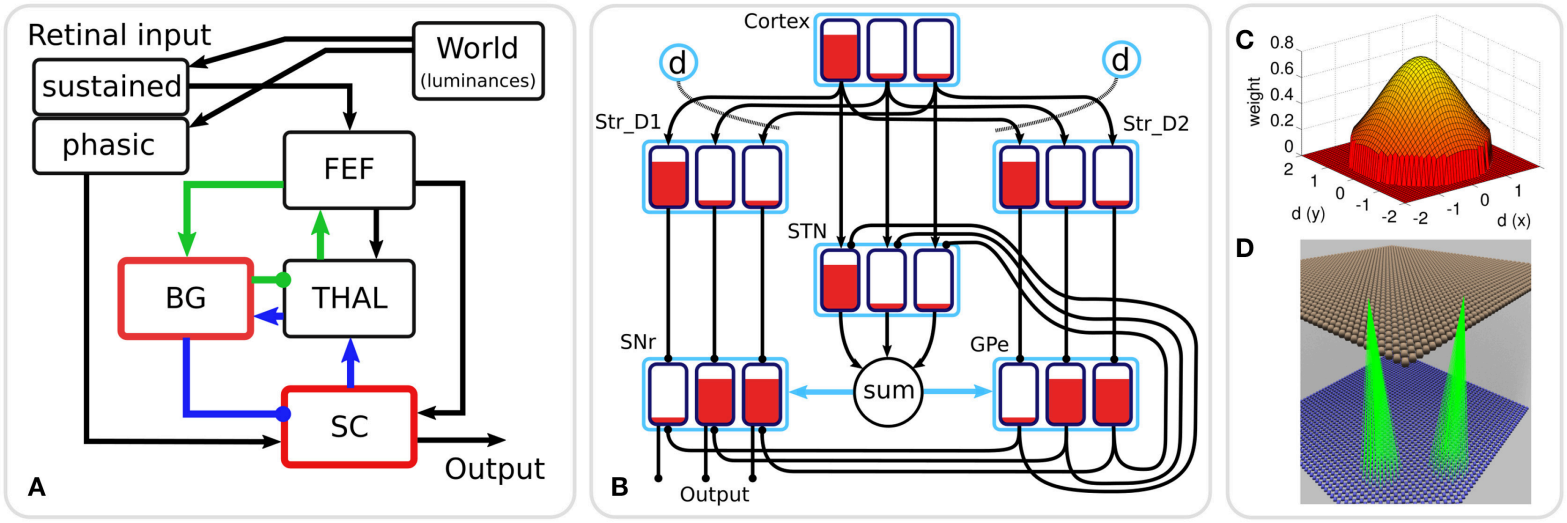
**(A)** The macroscopic architecture of the Cope-Chambers model. The main nuclei modeled as brain systems are: basal ganglia (BG), frontal eye fields (FEF), thalamus (THAL) and superior colliculus (SC). The retinal input is presented via non-biologically accurate retinal populations. The loops through basal ganglia, which define the architecture, are shown with colored lines: the cortical loop (through FEF and THAL) in green and the sub-cortical loop (through SC and THAL) in blue. Connections with arrowheads indicate excitatory connections, those with circles are inhibitory. A red border indicates that the box represents a sub-system of two or more populations; a black box indicates (at least, within the context of the model) a single neural population. The BG box is expanded in: **(B)** The basal ganglia model component. This shows a basal ganglia comprising striatum (Str_D1 & Str_D2), subthalamic nucleus (STN), globus pallidum externum (GPe) and substantia nigra reticulata (SNr). The model has three action channels shown as black boxes within each blue population border. Three channels of cortical input to the BG are also depicted. Red indicates the activation level of a given channel, helping to illustrate the selection mechanism. For example, the channel indicated by the leftmost bar has a high salience (cortical input) and excites activity in Str_D1 which then inhibits the leftmost bar in SNr. The diffuse projection from STN is equivalent to summing its projections channel-wise, and then projecting the sum to all channels of its target populations (the blue arrows indicate that all channels of GPe and SNr are targeted by the connection). Dopaminergic modulation of the inputs to the striatum are indicated by the blue circles labeled “d” and the dotted lines. The SNr sends inhibitory output projections to its targets. **(C)** 2D Gaussian weights. The “GaussianKernel” connectivity pattern is based on the in-plane component of the displacement between the location of a neural element in one layer, and the location of a target neural element in the target layer for the connection. The potential weight of the connection is given by a 2D Gaussian function, which is maximum for the target neuron which exactly corresponds to the source neuron, and drops down for target neurons which are horizontally displaced from the source neuron. A threshold is applied to avoid a computationally expensive all-to-all connectivity (with most of these connections having negligible weight values). If the weight is non-zero, then a connection is made from source to target, otherwise no connection is made. **(D)** Gaussian connectivity. This image shows connectivity (green rays) from two source neurons (in Str_D2, brown spheres) to target neurons (in GPe, blue spheres). The circular connectivity pattern is seen. This does not show the weight *values*, which reduce “toward the edge of the circle” and follow the relationship shown in **(C)**.

Figure 1A shows the macroscopic architecture of the Cope-Chambers model. The figure shows the relationships between the retinal input populations, the FEF, the populations comprising the BG sub-system (the red border indicates that the box represents a number of populations as a sub-system), the thalamus and the superior colliculus. Excitatory connections are indicated with arrowheads; inhibitory connections with circles in place of the arrowheads. The blue and green connection lines indicate two thalamo-basal ganglia loops, one cortical loop through FEF (green), the other a sub-cortical loop through SC. It is important to note that although they are given different colors in the diagram, these loops are in no way independent, with loop activity combining both in thalamus and in the basal ganglia and a direct excitatory, feed-forward connection from FEF to SC.

The basal ganglia sub-system is the most complex component of the Cope-Chambers model. The BG model is based on previous work (Gurney et al., 2001a,b) and is referred to as the GPR model. The GPR model incorporates the following main components of the primate BG (Mink, 1996; Wickens, 1997): (i) The striatum (the main input station to the BG) which is divided into two iterdigitated populations of projection neurons expressing primarily D1 or D2-type dopaminergic receptors (named Str_D1 and Str_D2); (ii) The subthalamic nucleus (STN); (iii) the external segment of the globus pallidus (GPe); (iv) the output nucleus relevant for saccadic control—the substantia nigra pars reticulata (SNr) (Hikosaka et al., 2000).

The connectivity of the GPR model (Figure 1B) is constrained by the known anatomy and physiology of the BG (Bolam et al., 2000). Physiologically, the only source of glutamate within the BG is the STN, whose projections are therefore excitatory; all other nuclei have GABAergic projection neurons and are therefore inhibitory. The cortex sends glutamatergic projections to both the Str_D1 striatal population, which projects preferentially to the SNr, and to Str_D2, which projects primarily to GPe (Gerfen et al., 1990). The cortex also projects to the STN, which sends diffuse projections to the SNr and GPe (Parent and Hazrati, 1993). The GPe projects to the SNr and also projects back to the STN, completing a GPe–STN loop.

The GPR model is arranged into “action channels”; Figure 1B shows an example network containing three channels. It is between these channels that competition occurs, with the winning channel succeeding in reducing activity in the output nucleus, SNr, and thereby disinhibiting its target. The complete connectivity pattern for this small network is shown in Figure 1B; the left channel in cortex innervates the left channels of Str_D1, STN and Str_D2. Connections are one-to-one, so it follows that the middle channel of cortex innervates the middle channels of STN and the striatal populations and the right channel of cortex innervates right channels in striatum and STN. Striatal population channels also inhibit SNr and GPe on a one-to-one basis and GPe feeds inhibition to SNr and STN in a one-to-one manner. The outputs from STN however are not one-to-one. The output from all channels of STN is summed together and then the sum is fed into each channel of SNr and GPe. This models the diffuse excitation from STN which has been observed in the BG (Parent and Hazrati, 1993).

Within the BG, there are several mechanisms supporting competitive processing for selecting channels whose inhibitory output should be reduced. The selection mechanism of the GPR model is the “off-center, on-surround” scheme proposed by Mink and Thach (1993). The “on-surround” is provided by diffuse, excitatory projections from the STN to the SNr. Focussed inhibition from the Str_D1 neurons in striatum contributes the “off-center” part of the mechanism. This arrangement leads to selection behavior via a release of target inhibition, since channels that have strong salience (input) have weak output at the level of SNr, and channels with weak salience have enhanced output.

The GPe is not included in the center-surround circuit described above, but still plays a key rôle in selection. Operating alone, the Str_D1/STN/SNr circuit can suffer from the following problem: if the input for all channels is relatively high, then the diffuse projection from STN, which effectively supplies a sum of *all* of the STN inputs to each channel in SNr, will provide so much excitation that Str_D1 may become unable to inhibit one of the channels in SNr and selection may become impossible. Gurney et al. (2001a,b) showed that the inhibitory feedback from GPe to STN acts as an “automatic gain control” to help prevent this from occurring.

At the neuronal level, the STN, GPe and SNr have tonic output activity (DeLong et al., 1985; Chevalier and Deniau, 1990; Kita and Kitai, 1991). This is modeled using piecewise linear output functions with zero offsets, *c* (see Equation 6) but with noise added to the input. In striatum, Str_D1 and Str_D2 have positive offset *c*, mimicking the so-called “down-state” of medium spiny neurons which have a resting potential far below spiking threshold and require co-ordinated input to generate action potentials (Wilson and Kawaguchi, 1996). In addition, the Str_D1 and Str_D2 neurons are influenced by dopamine in different ways; facilitating cortico-striatal transmission at medium spiny neurons with D1 receptors (Gonon, 1997; Hernández-López et al., 1997) and reducing transmission at those with D2 receptors (Delgado et al., 1999). These effects are modeled using a dopamine parameter, *d*, which modulates the input activations $a_{in}^{D1}$ and $a_{in}^{D2}$ as:

(1) $$a_{in}^{D1}=(0.2+d)A$$

(2) $$a_{in}^{D2}=(1-d)A$$

where *A* is the input activation (see also Equations 10, 11). For the “normal, healthy” value for *d* of 0.7, Str_D1 activation is relatively enhanced (0.9*A*); Str_D2 activation is one third of this value (0.3*A*). The major effect of this difference in the relative strength of the activity in Str_D1 vs. Str_D2 is simply that a change in the level of activity in Str_D1 affects the off-center, on-surround mechanism. The effect of varying the input into Str_D2 is much more subtle, with only a small change in the amount of inhibition fed from GPe into STN (via a focussed, one-to-one connection) being affected by the change, along with a small change in the inhibition fed into SNr from GPe (also via a one-to-one connection). It is not possible to ascribe the dynamic effect of the dopamine parameter to any single population, because the activity is recurrently connected through multiple loops. Thus, a line of reasoning such as “reduced activity for a luminance in Str_D2 will lead to less inhibition in that region in GPe, which means that there will be higher activity there, and hence more inhibition for that region passed to STN leading us to expect less activity in STN” is verified by running a suitable simulation with the model, but the effect is small. Note that the effect of dopamine in the model is only to modulate the strength of cortico-striatal synapses; no learning is modeled and so the significance of dopamine as a prediction error signal is outside the scope of the current work.

**Figure 2:**
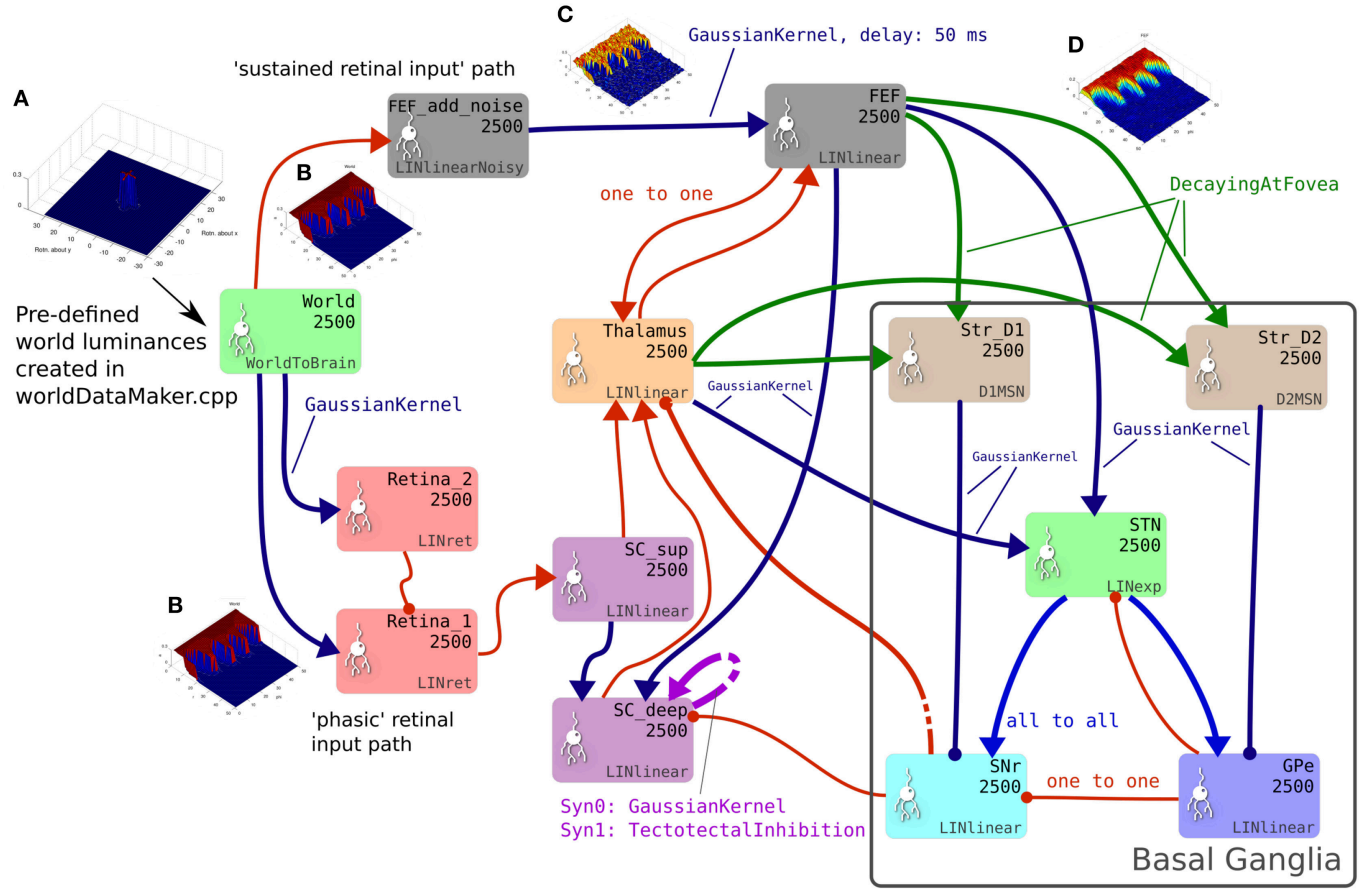
The brain model. This is the SpineCreator “network layer” view of the model. Each box represents a neural population with 2,500 elements, arranged in a 50 × 50 grid. The SpineML component name is printed on the bottom right corner of each population box and the population name is at the top. The overall connectivity between populations is represented by the projection arrows with the color indicating the connectivity scheme (one-to-one connections are red, Gaussian kernel connections are dark blue and so on). Excitatory connections have arrowheads and inhibitory connections have circles, although for full details of the behavior of the connections, the weight-update and post-synapse component associated with each connection must be referred to. Briefly, the model comprises a *World* population, into which a retinotopically organized view of the world is introduced. This information is passed into cortical populations (FEF) and subcortical populations (SC) via a simple model of the retina. These feed a cortico-thalamo-basal ganglia loop, which selects which region of the deep layer of superior colliculus should be disinhibited, allowing activity to build up therein. The five populations comprising the basal ganglia are enclosed in a gray outline. Note that substantia nigra pars compacta is not modeled here, instead the level of dopamine in the striatum is set via a parameter in the Str_D1 and Str_D2 populations. **(A)** Fixation cross input in Cartesian eye frame. **(B)** Fixation cross input in retinotopic co-ordinates. **(C)** Fixation cross with noise added. **(D)** After blurring, input is passed to BG populations.

The GPR model in Figure 1B has only three channels, with the focussed inhibition from striatum to SNr and GPe defined by a simple one-to-one scheme. The action channels represent discrete, incompatible motor action choices. In the oculomotor model, an action channel represents the end-point of saccade, and the competition carried out in the basal ganglia is between potential saccade end-points. However, eye movements have a *continuous* end-point space; the eye can rotate to any orientation within its biomechanically permissible range. Some end-points within this range are mutually exclusive—it's not possible to look to the left and to the right simultaneously—but *nearby* end-points are not necessarily incompatible. A small enough error in the end-point of a saccade will not prevent the eye from foveating on a target as the foveal region of high visual acuity is not infinitesimally small. To cope with this requirement, the populations within the oculomotor basal ganglia are conceived of as two-dimensional topographic grids of leaky integrator neural elements. Activity in each element corresponds to a spatial location in the visual field. Neighboring elements correspond to locations which are close to each other in the visual field. Focussed one-to-one projections in the GPR model are replaced by projective fields with many weighted connections. Specifically, each unit in Str_D1 projects to a counterpart SNr_*j*_ in SNr with some weight *w*_*max*_, but also connects to neighboring nodes in SNr with a weight given by *w*_*max*_.*G*(*d*), where *G*(*d*) is a circularly symmetric, 2D-Gaussian which is a function of distance *d* from SNr_*j*_ (Figures 1C,D). A similar scheme applies for the connectivity from Str_D2 to GPe and for a number of the other connections in the Cope-Chambers model; in the SpineML implementation of the model, this connectivity scheme is named “GaussianKernel.” Figure 2 shows a schematic of the SpineML implementation of the model, based on a diagram produced by SpineCreator. Populations for Str_D1, Str_D2, STN, SNr and GPe are shown within the “Basal Ganglia” box. Input comes into the model via the “World” population and the output population is SC_deep. Compare this diagram with Figures 1A,B. Figure 2 expands the “SC,” “BG,” and “slow retinal” boxes from Figure 1A.

The frontal eye fields (FEF) are a key cortical area for the generation of saccadic eye movements (Robinson and Fuchs, 1969; Bruce and Goldberg, 1985; Hikosaka et al., 2000; Tehovnik et al., 2000). Saccadic targets are retinotopically mapped on its surface (Robinson and Fuchs, 1969; Bruce and Goldberg, 1985; Sabes et al., 2002), and increased neural activity at a location on the map precedes a saccade to that location. Importantly, the FEF is also associated with visual decision making (Schall et al., 1995; Thompson and Bichot, 2005; Monosov et al., 2008; Cohen et al., 2009). Thus, in a saccade choice, increased FEF activity is predictive of the eye movement whether correct or incorrect (Thompson et al., 2005), rather than of the correct response.

FEF neurons can be divided into three functional groups, related to whether their activity corresponds with visual stimuli, motor action, or both (Segraves and Goldberg, 1987). The Cope-Chambers model simplifies this categorization using a single layer of 50 by 50 units representing the mean of all three groups. This layer therefore responds to both visual stimuli and the buildup of activity associated with motor (saccadic) action. The retina provides a persistent luminance signal into the FEF through the dorsal visual pathway (Ungerleider and Mishkin, 1982) which is abbreviated in this model to a direct connection with delay and noise.

The FEF provides input into the BG (Saint-Cyr et al., 1990) (to Str_D1, Str_D2 and STN) which, in turn, projects back to thalamus in a retinotopically organized way (Lynch et al., 1994; Middleton and Strick, 2000). In addition, the thalamic targets of this path are regions with strong reciprocal connections to the FEF (McFarland and Haber, 2002). In this way, the FEF forms channel-based loops through basal ganglia of the kind described above. Such circuits formed the basis of the model of Humphries and Gurney (2002). The thalamo-cortical loop may be thought of as an integrator of information, whose gain is modulated by inhibition from basal ganglia (Chambers et al., 2011; Cope and Gurney, 2011).

The superior colliculus (SC) is a sub-cortical nucleus which also plays a critical rôle in the generation of saccades (Hikosaka and Wurtz, 1983). Both FEF and SC have direct connections to the saccadic burst generator (SBG, see section 2.3). If either is lesioned, the other can direct gaze, following a period of adjustment (Latto, 1977), albeit with some persistent deficits. The SC is also a direct target of output from the SNr (Jayaraman et al., 1977; Jiang et al., 2003) and can be influenced by the action selection mechanisms of the BG. In particular, it forms a loop with BG, but unlike its cortical counterpart in FEF, the input to basal ganglia comes via the thalamus (Figure 1A, blue arrows).

While the SC has seven alternating cell- and fiber-rich layers (Wurtz and Albano, 1980), in most cases these are categorized as the “superficial” and “deep” layers, which have significantly different response properties. Cells in the superficial layers, which receive input from the retina, are mainly visually responsive, with a preferred response to phasic events (luminance onsets and offsets) and movement on the visual field (Goldberg and Wurtz, 1972). In contrast, cells in the deep layers receive multi-modal input, including inhibitory input from the output structures of the BG (Jayaraman et al., 1977), and are directly involved in the generation of saccadic eye movements. Saccade related activity in the deep layers appears to generate saccades through “population coding,” with a weighted sum of activity across the retinotopy of SC determining the saccade target (Mays and Sparks, 1980; Lee et al., 1988; van Opstal and van Gisbergen, 1990). The deep layers of SC receive input from the FEF in a topographic manner (Stanton et al., 1988a; Sommer and Wurtz, 2000).

The SC in the Cope-Chambers model is based on the the model described in Arai and Keller (2005), with the difference that the SNr input to the SC is generated by the BG model, rather than being hand-crafted. The SC model has a superficial and a deep layer, each of which is a 2-D array of 50 by 50 leaky integrator units arranged in the same retinotopic manner as the FEF (Wurtz and Albano, 1980).

**Figure 3:**
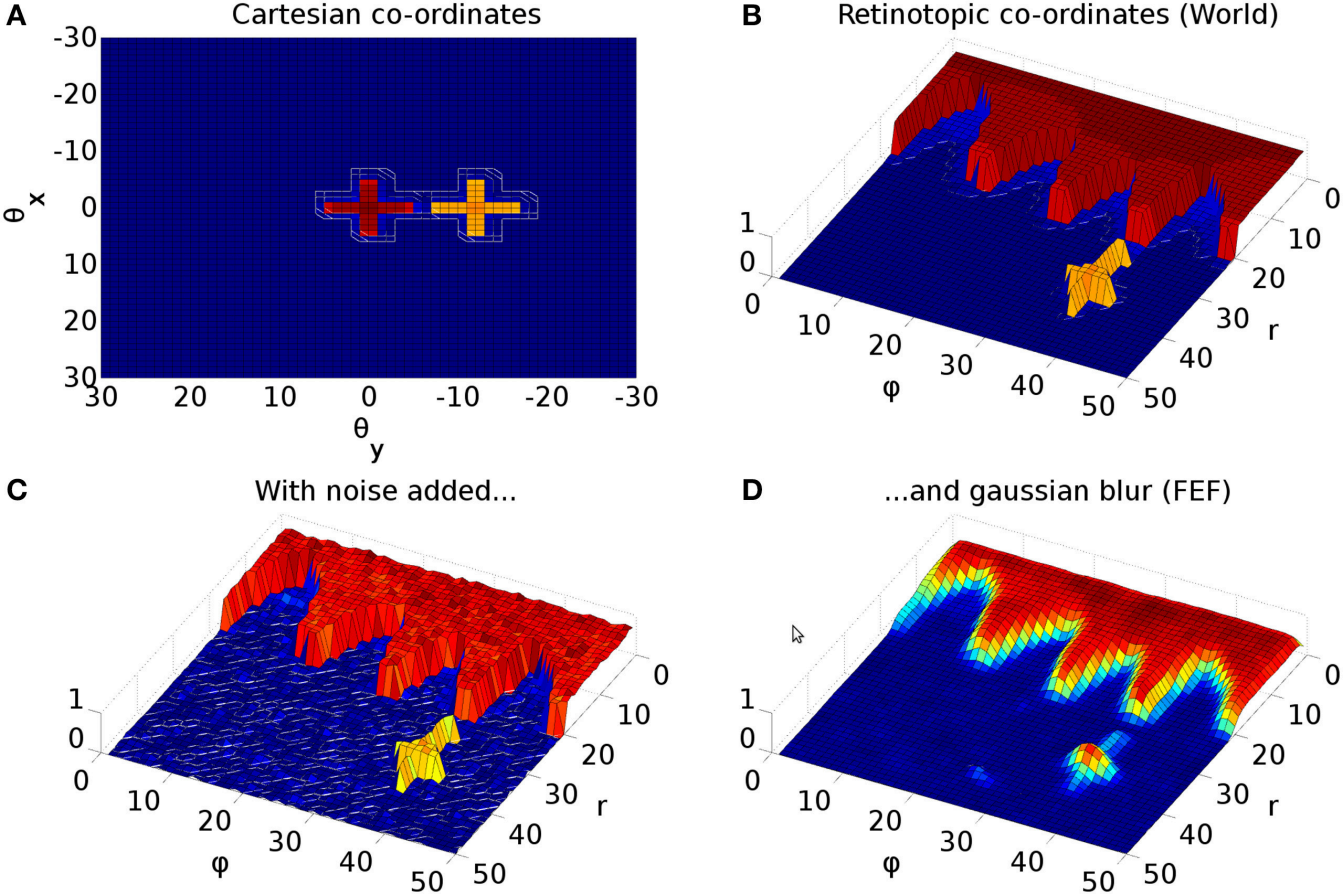
Representative mapping from eye's frame of reference in Cartesian co-ordinates to retinotopic co-ordinates. **(A)** The mapping of luminances in the eye's frame of reference. The world input is pre-defined by a JSON configuration file. Luminance position, size and shape can be defined in this file, along with the times at which luminances appear and disappear. The worldDataMaker.cpp code computes the locations of the luminances in the eye's frame of reference, given its rotational state. It also computes a 2D Gaussian convolution of the luminances. Here, there are two cross shaped luminances spanning 10°, one of value 0.8 at the fixation point (0,0) and one of value 0.5 at a peripheral position (0, −12°). Note that these crosses have the same “bar width” of 2° as the crosses used in the simulations, but their span of 10° is greater than the 6° used in the simulations, to make these images clearer. **(B)** The locations of the luminances in the eye's frame of reference are then converted into retinotopic co-ordinates, with centrally located luminances being represented at low values of *r* and more peripheral luminances having higher values of *r*. ϕ encodes rotational angle: 1 and 50 encode upward movement; 13.5 is left; 26 is down; 38.5 is right. The output of the World component is fed into FEF_add_noise and into the retinal neuron populations. The color map makes it possible to distinguish between the two crosses. **(C)** The FEF_add_noise populations adds a level of noise to the signal representing processing of the signal in visual cortex. **(D)** A Gaussian projection from FEF_add_noise to FEF further blurs the activity in FEF. FEF is the input to the basal ganglia and one input to superior colliculus.

The Cope-Chambers model incorporates a special connectivity pattern for visual input to the BG via cortical (FEF) and sub-cortical (thalamus) pathways. Due to the retinotopic mapping (section 2.2.2), foveal luminances deliver a strong signal to the BG; roughly one third of the map is activated for the foveal targets used in this work (Figure 3, red cross). This makes it virtually impossible for a peripheral target (Figure 3, yellow cross) to win selection in the BG. Even if the peripheral target competed successfully to generate a saccade, this process would cause a significant delay, leading to latencies much larger than those observed experimentally. To overcome this problem, the Cope-Chambers model incorporates a mechanism in which the synaptic strength of connections between FEF, thalamus and striatum are reduced close to the fovea according to a shifted hyperbolic tangent. This connection is named “DecayingAtFovea” in the SpineML implementation and follows a modified sigmoidal curve rather than tanh. In either case, the relation is “S-shaped” and normalized to the range [0 1]. Far from the fovea (where the S-shaped curve has the value ≈1), the connectivity pattern looks almost identical to a one-to-one connection.

Input to the Cope-Chambers model is provided through a simple retina model which directly samples from a larger “world array” of pixel values. In the current model, the input for the retina is named “World” and is the retinotopic projection (Figure 2B) of the eye's field of view of the world (Figure 2A) and the luminant targets therein. The raw input in “World” is fed into a population which adds noise, and then via a delayed connection to FEF (the sustained retinal input path), to simulate processing through the dorsal visual stream. It is also fed, without substantial delay, into two leaky integrator layers (Retina_1 and Retina_2) with different time constants, with the more slowly reacting layer (Retina_2) inhibiting its faster counterpart. The faster layer responds quickly to the appearance of a prolonged stimulus before it is inhibited by the slow layer, forming a phasic response to stimulus onset. The mechanism ensures that phasic rather than tonic responses arrive at the superficial SC from the retina.

The output of the Cope-Chambers model is determined by the activity in the SC_deep population. The activity in SC_deep is first transformed from retinotopic co-ordinates into Cartesian co-ordinates. The centroid of the activity is then computed. The position of this centroid in the Cartesian frame determines the saccadic end-point. The current model differs in that it does not compute a centroid, instead feeding the SC_deep activity into the saccadic burst generator.

The Cope-Chambers model was parameterized by tuning the model to perform a prosaccade task in which a central luminance point was fixated by the model. After a fixed duration, the fixation point was extinguished and a target point of fixed luminance was presented. The model was tuned so that the latency between the presentation of the target and the initiation of an eye movement matched experimental data (Reulen, 1984), while also matching the electrophysiological evidence of activity in a variety of brain regions. The tuning of the BG model attempted to preserve as closely as possible the weights used in the original paper (Gurney et al., 2001b). Further details on the parameterization of the Cope-Chambers model are given in Cope et al. (2017).

#### 2.2.1. Components

With the exceptions of the World and FEF_add_noise populations, each neural element represents an activation; the activation is governed by a first order differential equation specified in a *SpineML component*. SpineML, which will be outlined in section 2.5, provides a means to mathematically define the five distinct components in use in the brain model.

The *LINlinear* component, which is used in FEF, Thalamus, SC, SNr and GPe populations, governs the activation *a* with a first order leaky integrator differential equation:

(3) $$\dot{a}=\frac{1}{\tau }(a_{in}-a)$$

where τ is the time constant for the neural activation and *a*_*in*_ is the input to the neural element. *a*_*in*_ is defined by an activation input and a shunting inhibition input according to:

(4) $$a_{in}=A(1-s_{a})+\alpha R_{N}$$

Here, *A* is the activation input and *s*_*a*_ is the shunting inhibition state variable whose value is related to the shunting input, *S* by

(5) $$s_{a}=\{\begin{array}{ll} S & S\leq 1\\ 1 & S>1 \end{array}$$

*R*_*N*_ is a random number drawn from a standard normal distribution (σ = 1, μ = 0) and introduces noise to the activation of the neural element, with the parameter α controlling the noise amplitude.

The output, *y*, of LINlinear is related to the activation *a* by the piecewise linear transfer function

(6) $$y(a)=\{\begin{array}{ll} 0 & a<c\\ a-c & c\leq a\leq 1+c\\ 1 & a>1+c \end{array}$$

where *c* is a parameter defining the offset of the transfer function. If *c* < 0, then for zero activation (*a* = 0), the output will be positive. This simulates the effect of a neural population having tonic firing. If *c* > 0 then the output will be zero until the activation exceeds *c*, simulating neurons which only fire when driven by excitatory input.

The *LINret* component used for the retinal populations is similar to the LINlinear component, but with no intrinsic noise and no shunting inhibitory input. It has a neural input which is identical to the activation input *A*:

(7) $$a_{in}=A$$

The *LINexp* component is a leaky integrator with an exponential transfer function. It shares the same differential equation with LINlinear, but has a different input equation and a different output transfer function. It has the following equation for the neural element input *a*_*in*_:

(8) $$a_{in}=[A+N(a-V_{r}^{-})](1-S)+0.01R_{N}$$

where *A* is the activation input and *N* is an input which is modulated by $V_{r}^{-}$, a reversal potential, and *a*, the current activation of the element. These inputs are summed and then reduced by a factor which is dependent on *S*, the shunting input. As in LINlinear, *R*_*N*_ introduces normally distributed noise to the element.

The output, *y*, of the LINexp component is given by

(9) $$y(a)=\{\begin{array}{ll} {\text{e}}^{a}-0.9 & {\text{e}}^{a}\leq 1+0.9\\ 1 & {\text{e}}^{a}>1+0.9 \end{array}$$

This component is used in the subthalamic nucleus (STN) population, as it gives a more physiologically accurate f-I behavior (Bevan and Wilson, 1999; Hallworth et al., 2003; Wilson, 2004) which has been shown to allow the mapping of the basal ganglia network architecture onto an optimal decision making model (Bogacz and Gurney, 2007).

The *D1MSN* and *D2MSN* components are both leaky integrators, similar to LINlinear. They differ in that they have no shunting inhibition. They are used to model medium spiny neuron (MSN) populations in the striatum. As they model the fact that most MSN neurons fall into two groups; those expressing D1 dopamine receptors and those expressing D2 receptors, they have a dopamine parameter that modulates the input activation, so that their equations for *a*_*in*_ are thus:

(10) $$a_{in}^{D1}=(0.2+d)A+0.01R_{N}$$

(11) $$a_{in}^{D2}=(1-d)A+0.01R_{N}$$

where *d* is the dopamine parameter. Varying dopamine from 0 to 1 enhances the activation in the D1 model, whereas it decreases the activation of the D2 model elements, in line with experimental observations (Gonon, 1997; Harsing and Zigmond, 1997). Note that the equation for $a_{in}^{D1}$ differs from that used in the Cope-Chambers model, for which the cortico-striatal weights are multiplied by (1+*d*) rather than (0.2+*d*). A typical value of *d* is 0.7.

In the typical components given above, the value of the activation *A* (and where relevant, the shunting input, *S*) is determined by summing the weighted inputs to the population:

(12) $$A=\sum_{i}w_{i}^{act}x_{i}^{act}$$

(13) $$S=\sum_{i}w_{i}^{sh}x_{i}^{sh}$$

$w_{i}^{act}$ and $w_{i}^{sh}$ are, respectively, the weights of the *i*^*th*^ activation or shunting connections received by the component; $x_{i}^{act}$ and $x_{i}^{sh}$ are the signals input to the activation and shunting connections.

#### 2.2.2. Population activity and retinotopic mapping

Each population of 2,500 neural elements was arranged in a 50 by 50 grid, with positions on the grid representing a retinotopic mapping similar to that found empirically both in the superior colliculus (Ottes et al., 1986) and in visual cortex (Schwartz, 1980) and assumed in this work to persist throughout the oculomotor system.

In a retinotopic mapping, the Cartesian co-ordinates of the light-sensitive cells in the retina, whose density varies with distance from the fovea, are transformed into the Cartesian co-ordinates of the correspondingly active cells on the colliculus. The mapping ensures that an even density of cells can be maintained in the colliculus, but ensures that a group of adjoining, active, retinal neurons will always activate an adjoining group of neurons on the collicular surface.

The mapping turns out to resemble polar co-ordinates. That is, one axis of the collicular surface specifies the eccentricity of a retinal location (how far it is from the fovea) and the second axis specifies the rotational angle of the retinal location; we therefore use the convention of referring to the eccentricity axis on the colliculus as *r* and the rotation axis as ϕ.

The *cortical magnification factor*, *M*(*r*), gives the relationship between the radial eccentricity *r* and the retinal neural density. As in Cope et al. (2017), we use a first-order approximation of the form for *M*(*r*) given in Rovamo and Virsu (1979):

(14) $$M(r)=\frac{M_{f}}{1+\frac{r}{E_{2}}}$$

The foveal magnification, *M*_*f*_, is the magnification of the most central region of the retina and has a value in the human of about 7.8 mm/° (Rovamo and Virsu, 1979).

In our model, *M*_*f*_ is related to *W*_*nfs*_, the width of the retinotopic neural field, *W*_*fov*_, the width of the eye's field of view and *E*_2_, the eccentricity at which the retinal density has halved by:

(15) $$M_{f}=\frac{W_{nfs}}{E_{2}\;\ln\left(\frac{W_{fov}}{2E_{2}}+1\right)}$$

Here, *W*_*nfs*_ is 50 (the side length of the 50 × 50 grid) and *W*_*fov*_ is set to 61°, a reduction from the biologically accurate 150° due to the small number of neurons in the retinotopic neural field. *E*_2_ is 2.5 (Slotnick et al., 2001; Cope et al., 2017).

The mapping from the retinotopic co-ordinates in the brain to rotational co-ordinates of the stimulus/response was written down by Schwartz (1977, 1980) for measurements of striate cortex (visual stimulus to electrophysiological response—Talbot and Marshall, 1941; Daniel and Whitteridge, 1961) and by Ottes et al. (1986) for superior colliculus data (electrophysiological SC stimulus to eye movement response—Robinson, 1972). We used the following statement of this mapping to introduce stimuli into the “World” input population of the brain model:

(16) $$\phi =\frac{W_{nfs}}{2\pi }\arctan\left(\frac{\theta_{y}^{t}}{\theta_{x}^{t}}\right)$$

(17) $$r=M_{f}E_{2}\ln\left(\frac{1}{E_{2}}\sqrt{\theta_{x}^{t2}+\theta_{y}^{t2}}+1\right)$$

Note that we use *r* and ϕ as the co-ordinates on the “collicular surface.” Schwartz uses *r* and ϕ as the polar co-ordinates of the retinal stimulus; Ottes et al. use *r* and ϕ as polar co-ordinates for the eye movement response; both use *u* and *v* as the Cartesian co-ordinates of the neural map. We use $\theta_{x}^{t}$ and $\theta_{y}^{t}$ to give Euler rotations for the retinal target stimulus. Note also that the form of Equations (16) and (17) is slightly different from that given in Ottes et al. (1986) because our $\theta_{x}^{t}$ and $\theta_{y}^{t}$ are not the polar co-ordinates used in that work.

The mapping encompases the entire visual field; the value of ϕ is allowed to vary from 0° to 360° along its axis. Effectively, the two contralateral colliculi found in the biology are incorporated into a single, square map, avoiding the need to carry out the kind of “colliculus gluing” described in Tabareau et al. (2007).

It is straightforward to show that the reverse mapping is given by:

(18) $$\theta_{x}=E_{2}\left(e^{\frac{r}{M_{f}E_{2}}}-1\right).\cos\left(\frac{2\pi \phi }{W_{nfs}}\right)$$

(19) $$\theta_{y}=E_{2}\left(e^{\frac{r}{M_{f}E_{2}}}-1\right).\sin\left(\frac{2\pi \phi }{W_{nfs}}\right)$$

where we have dropped the *t* superscript on θ_*x*_ & θ_*y*_, as these equations transform a collicular location into rotations of the eye.

Figure 3 shows the result of the mapping for a view of two cross-shaped luminances. One cross illuminates the fovea, which results in a large comb-shape of activity. The more peripheral cross produces (in FEF) an indistinct object centered at a larger value of *r*.

#### 2.2.3. Network

Briefly, the model consists of input from the World population (see Figure 2, green population box) producing activity in an “express” pathway to superior colliculus (purple) and simultaneously in cortex, represented here by the FEF population (gray boxes in Figure 2). The express pathway causes short latency activity in the superficial superior colliculus, which directly innervates the deeper layers of the superior colliculus (SC_deep). Activity in FEF generates firing in a thalamo-cortico-basal ganglia loop. The output of the basal ganglia is the substantia nigra pars reticulata (SNr) which tonically inhibits SC_deep. If a location of activity in FEF is able to dominate selection in the basal ganglia circuit, the corresponding location in SNr will dis-inhibit and activity will build up in SC_deep encoding the saccade end point.

Connections shown in red are one-to-one connections; dark blue projections indicate a connectivity pattern which “fans out” with a 2-D Gaussian kernel (Figure 1C); lighter blue connections from the STN to SNr and GPe are diffuse, all-to-all connections and projections colored green are one-to-one connections that decay toward the fovea so that foveal activity in FEF does not swamp the basal ganglia which would prevent peripheral luminances from ever being selected. Note that SC_deep contains two recurrent connections; one is excitatory, with a Gaussian kernel mapping and the other implements tecto-tectal inhibition, which increases the inhibition between activity in opposite hemispheres of the field of view (Gian and Jorge, 1981; Olivier et al., 2000) helping to resolve competition between saccades to the left and right. The tecto-tectal inhibitory connection is *not* present in the Cope-Chambers model. In all other respects the model is as described in Cope et al. (2017). We have not listed the parameters of the network in tabular form here, instead, the reader is referred to the SpineML declarative specification of the model from the link given in Supplemental Data. The easiest way to access this information is by using SpineCreator.

### 2.3. Brainstem model

**Figure 4:**
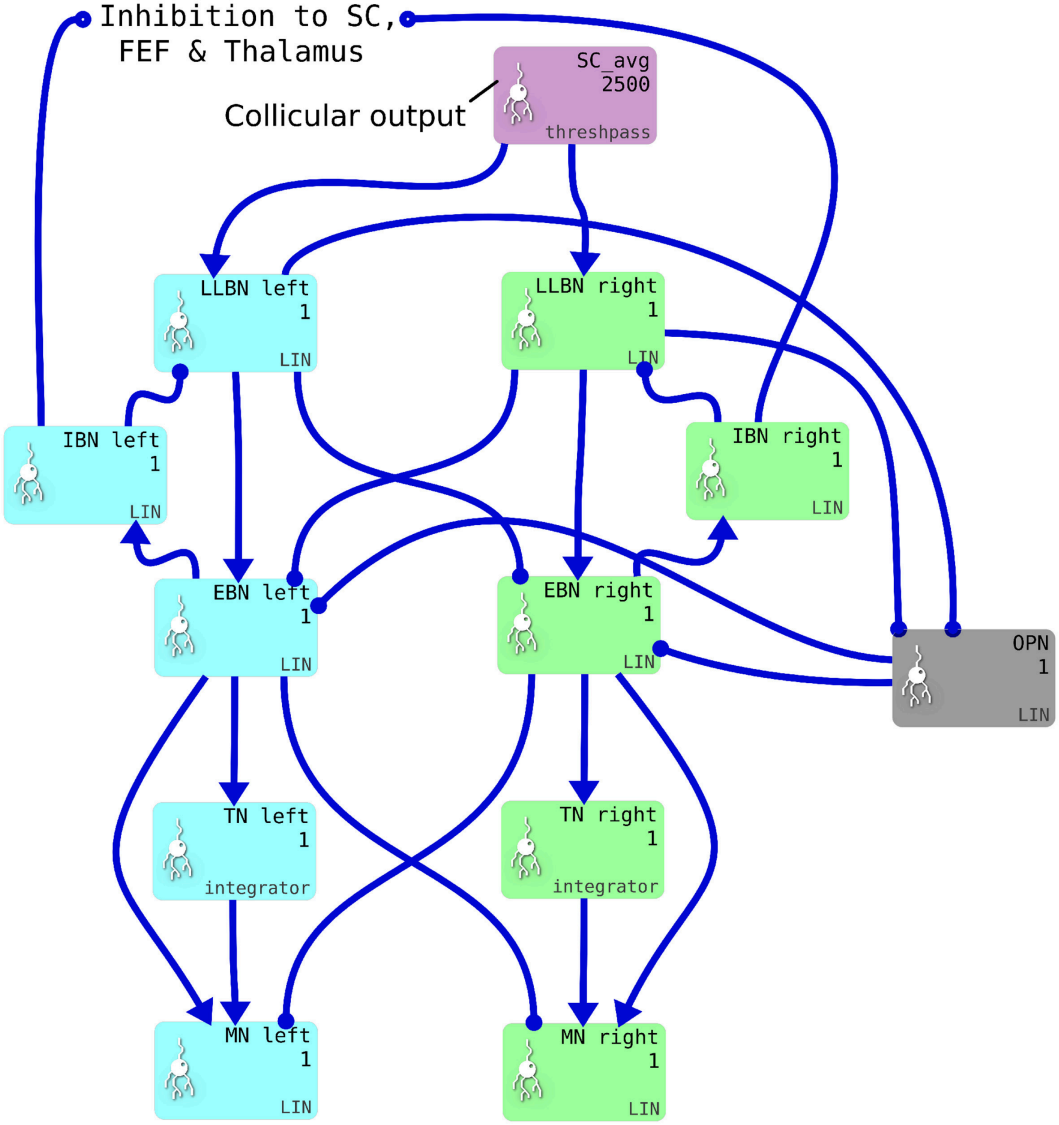
One pair of channels of the saccadic burst generator (SBG) for left (cyan) or right (green) movements. Collicular activity in SC_avg excites the channels via SBG weight maps, which are encoded as explicit lists of connection weights in the blue connection arrows from SC_avg to the LLBN populations. Each box represents a neural population and shows the population name, the number of neural elements (here 2,500 or 1) and the SpineML component name; *LIN* for Leaky integrator or *integrator*. Key: LLBN, Long lead burst neurons; IBN, Inhibitory burst neurons; OPN, Omnipause neurons; EBN, Excitatory burst neurons; TN, Tonic neurons; MN, Motoneurons.

We implemented a saccadic burst generator (SBG) based on the connectivity outlined in Gancarz and Grossberg (1998). The SBG network for two of the model's six channels is shown in Figure 4. In the brainstem model, we use the word “channel” to mean a set of populations of neurons which are involved in actuating a single extraocular muscle. SBG channels are arranged in pairs, actuating opposing muscles. There is one pair of channels which actuates the superior and inferior rectus muscles, causing vertical rotations of the eye in a roughly parasaggital plane (the eye moves up or down). Another pair actuates the lateral and medial rectus muscles, causing horizontal rotations of the eye. The third pair actuates the superior and inferior oblique muscles which contribute to vertical as well as oblique rotations. Activity from the output layer of superior colliculus (SC_avg) is fed into each channel, which sums the activity it receives and processes it in populations each of a single neural element representing all the neurons in that population. Each channel of the SBG functions to create the motor neuron activations that are required to accelerate the eye in a particular direction, then hold the eye in its new position against the returning force generated by the elastic properties of the muscles. The required motor neuron activations are therefore a combination of features: a brief burst of increased activity that accelerates the eye; followed by a period of activity that is less than the burst firing rate but higher than the tonic rate that exists when the eye is at the center. This holds the eye in its new position.

The SBG connectivity produces each of the these features separately, then sums them to create the desired “bump and tonic” activation time series. The input to the first population in the SBG, the long-lead burst neurons (LLBNs), is conceived as originating from one of the deep layers of the superior colliculus. The activity of the LLBNs are passed to excitatory burst neurons (EBNs) which, in turn, inhibit the LLBNs via the activity of the inhibitory burst neurons (IBNs). This feedback loop has a transmission delay, which allows activity to build up in the EBNs before the inhibition is activated and the activity is then reduced again. This mechanism generates the “bump.”

The generation of the “tonic” phase of the required time series is achieved simply by integrating the bump over time and multiplying by a some small gain factor. This is the function of the tonic neurons (TNs). The firing rate of the motor neuron defines the amount of force applied to the eye by that muscle. Thus, the integral of the “bump” defines how far the eye moves in that channel's direction. The gain and delay parameters in the LLBN-EBN-IBN-LLBN feedback loop therefore have to be tuned such that the endpoint of the saccade is reasonably accurate. Furthermore, the restoring force generated by the elasticity of the muscles is dependent on the radial distance. The value of the new tonic firing rate, after the “bump” is dependent on the end location of the eye. If the ratio between the EBN firing rate and the TN firing rate is not exactly correct, the eye will drift away from the saccade endpoint after the saccade has been completed. The EBN-TN connection strength is therefore tuned such that the TN firing rate yields a stable eye position across a range of eye eccentricities.

The omnipause neurons (OPNs) are tonically active and inhibit the EBNs. The activity of the OPNs is itself inhibited by activity in the LLBNs. The purpose of this arrangement is to ensure the eye does not move in response to neural noise.

Each mean activity of all the neurons in each SBG population (except the TNs) is defined by a single leaky integrator, first order differential equation.

(20) $$\frac{da}{dt}=\frac{1}{\tau }(y-a)$$

where *a* is the activation of the nucleus, and τ is the time constant of the nucleus. *y* is a piecewise linear function of the weighted sum of inputs to the nucleus and is given by

(21) $$y(IN)=\{\begin{array}{ll} 0 & IN\leq b\\ IN-b & b\leq IN\leq 1+b\\ 1 & IN\geq 1+b \end{array}$$

where *b* is the *IN* axis offset. *IN* is the weighted sum of inputs to the nucleus and is given by,

(22) $$IN=\sum_{m}^{M}w_{mn}a_{m}$$

where *a*_*m*_ is the activation of the *m*th afferent nucleus. *w*_*mn*_ is the connection strength between the *m*th afferent nucleus and the current nucleus. The activity of the TNs are defined as

(23) $$\frac{da}{dt}=\frac{1}{\tau }y$$

with the same piecewise linear transfer function as in the other SBG populations.

### 2.4. Biomechanical eye

The output signals of the brainstem's motoneuron (MN) populations are used to drive the biomechanical model. The MN output signal in each brainstem channel is normalized in the range [0 1] and represents the mean firing rate of the neurons that innervate the extraocular muscle for that channel. The biomechanics are used not only to get tangible feedback on the simulated saccades including motion trajectories, but to add one more modeling dimension related to the inertial properties of the eye plant including muscle properties.

The biomechanical eye model, implemented using the OpenSim framework (Seth et al., 2011), is anatomically represented by a sphere of uniform mass distribution. The diameter of the eye is 24 mm for adults, with small variations between individuals; the mass of the eye is 7.5 g. The eyeball is actuated by six extraocular muscles (EOMs). The EOMs are arranged in three pairs forming a cone inside the orbit with the apex being located inside the cranium in a tendonous ring called the annulus of Zinn. An important feature of the oculomotor system which greatly affects its overall behavior is the existence of dynamic EOM pulleys. Their role is to guide the pivot point of the EOMs. In our model, a pulley for each EOM has been modeled by a point on the orbit whose location depends on the current eye orientation.

**Figure 5:**
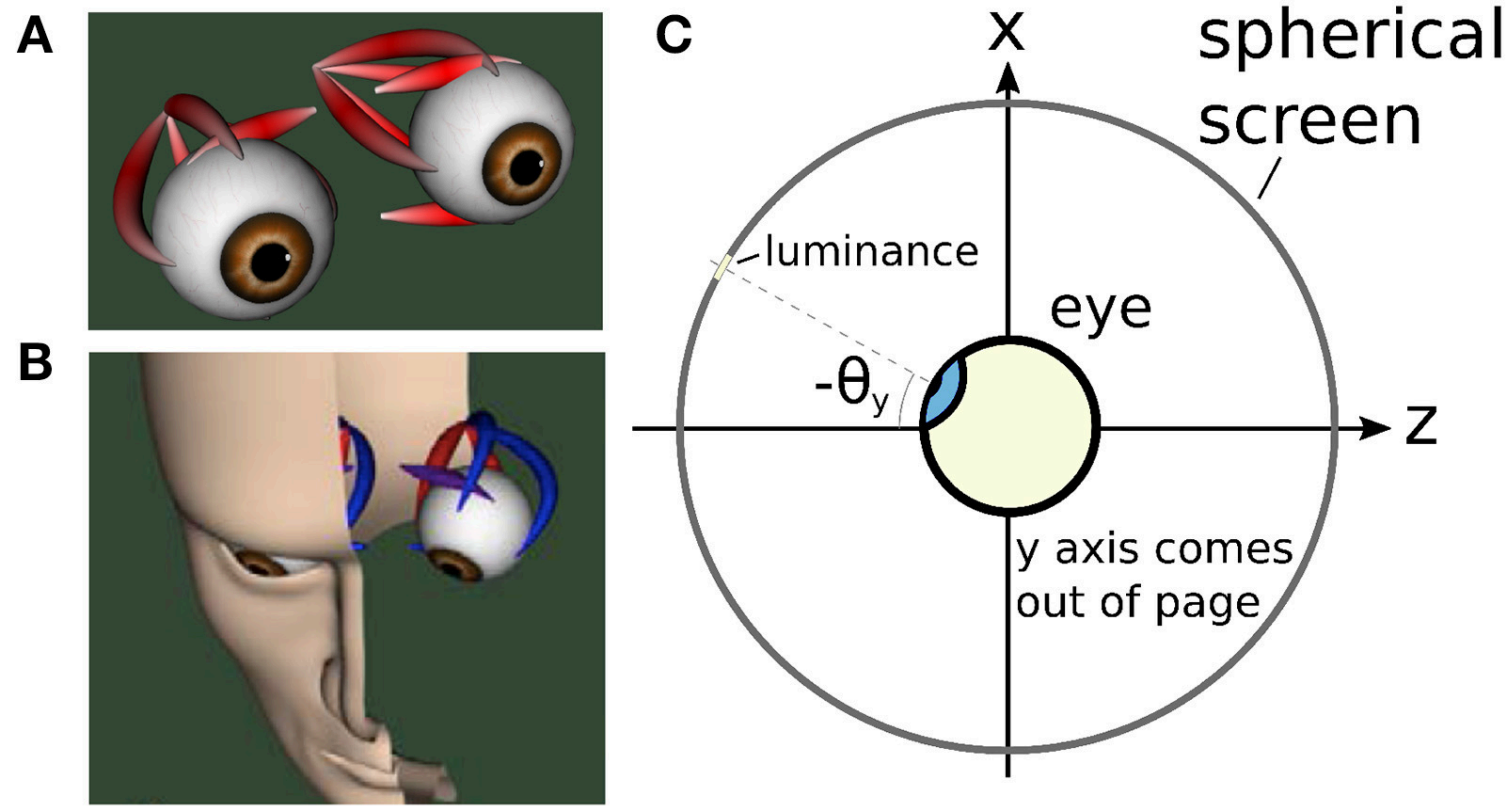
The biomechanical eye. **(A)** an OpenSim rendering of a pair of biomechanical eyes showing the positions of the extraocular muscles. Note that (i) volume visualization of muscles should not be confused with FEM muscle models; it is provided for user feedback purposes, i.e., shape and color change depending on the muscle activation, (ii) superior and inferior oblique are visualized up to their respective muscle pulleys. **(B)** OpenSim rendering of biomechanical eye within a head model. **(C)** Top-down schematic cross-sectional view of the biomechanical eye situated within a spherical screen, with a horizontal rotation toward a luminance at an angle of −θ_*y*_ about the *y* axis. The *y* axis points up, out of the page.

An illustration of the biomechanical eye model is given in Figure 5A, while Figure 5B depicts the head model used in the proposed framework. Figure 5C shows a cross-sectional view of the eye and the spherical screen on which targets were projected.

Two types of muscle models of different complexity are supported. The first models muscles using linear path actuators. This simplistic model of ideal muscles can be easily integrated with high level brain models. As described above the muscles are wrapped around the eye. The more complex model supported is based on the Thelen model (Thelen, 2003) that is also supported by OpenSim and implements Hill-type muscles. It includes realistic muscle wrapping geometric entities of the muscle fibers, while it accommodates for both activation and contraction dynamics. The dynamics of muscular forces can be split into: (1) The elasticity of the muscles. (2) A delay between the onset of the afferent excitatory signal and the actual muscle contraction, caused by the transmission time of the action potentials and by the necessary calcium release at the muscle fibers.

The force applied by EOMs is controlled by an excitatory signal supplied by motoneurons in the brainstem. The neural drive to produce a saccadic eye movement can be characterized by a pulse component to overcome the viscoelasticity of the orbital plant, a step component to stabilize the eye in the new position, and a slide component that models the gradual transition between the pulse and step.

Passive forces due to the fatty tissues inside the eye orbit also affect eye dynamics. Their role is critical in eliminating the influence of head and body movements. We incorporated a custom torque, **t**, which acts like a rotational spring-damper apparatus, resisting eyeball movements. It has elastic and viscous properties governed by **t** = −*K***R** − *C***U** where **R** is the eye's orientation and **U** is its angular velocity. *K* and *C* are constants. A fuller description of the biomechanical model can be found in Papapavlou and Moustakas (2014).

Finally, numerical integration of the biomechanical eye model is based on the Kutta-Merson integration method.

### 2.5. Model development framework

The Cope-Chambers model was originally developed to run on the BRAHMS model execution framework (Mitchinson et al., 2010; Mitchinson and James, 2015). To run a BRAHMS model, the researcher must develop *BRAHMS components* for the various neural elements. A BRAHMS component is a programmatically coded implementation of the behavior of the component. It may have an arbitrary number of inputs and outputs and may be written in C, C++, Python or MATLAB. The Cope-Chambers model components were hand written in C++ and MATLAB. A BRAHMS *SystemML* file describes how the different components connect together and how data is passed between them (Mitchinson et al., 2010). The main BRAHMS program first reads the SystemML file, then dynamically loads all the required components before executing the system.

In the current work, the Cope-Chambers model was reproduced using the declarative SpineML markup language (Cope and Richmond, 2014; Richmond et al., 2014), with the help of the graphical SpineML model editing software called SpineCreator (Cope et al., 2015, 2016a). SpineML, which is a development of the NineML specification (INCF Task Force on Multi-Scale Modeling, 2011), describes neural populations and their projections in a highly structured format in which neuron bodies, pre- and post-synapses are described in terms of *SpineML components*. These are similar to the components provided by BRAHMS, but in this case, the components are an XML description of the functionality of the component, rather than a programmatic implementation, with one XML file per component. A SpineML *network layer* file then describes which components are used in the model, and how they are connected together. Finally, a number of SpineML *experiment layer* files specify how the model described in the network layer can be executed. In the experiment layer, the execution duration and timestep can be specified, along with input conditions, connection lesions and component parameter updates. A description of SpineML is given in Richmond et al. (2014); the definitive definition is found in the schemas (Cope et al., 2014). SpineCreator, in its rôle as a graphical editor for the SpineML format, was used to generate the SpineML files describing the model. It was also used to generate the diagrams of the model.

As a declarative format for model specification, SpineML is agnostic about how the model is executed. A number of simulation engines can be utilized, including DAMSON (Richmond, 2015), GeNN (Nowotny, 2011; Nowotny et al., 2014) and BRAHMS (used here). The simulation engine incorporating BRAHMS is called SpineML_2_BRAHMS (Cope and James, 2015). SpineML_2_BRAHMS is a collection of XSLT stylesheets which first generate and compile C++ BRAHMS components (which implement a simple, Forward-Euler solver) from the SpineML component layer description files. SpineML_2_BRAHMS then uses the SpineML network and experiment layer files to generate a BRAHMS SystemML description of the model. Finally, SpineML_2_BRAHMS executes the model now described entirely as a BRAHMS system, via a call to the BRAHMS binary. A number of additional, hand-written components are present in SpineML_2_BRAHMS providing the inputs (constant inputs, time-varying inputs, etc) which the modeler specifies in the experiment layer.

**Figure 6:**
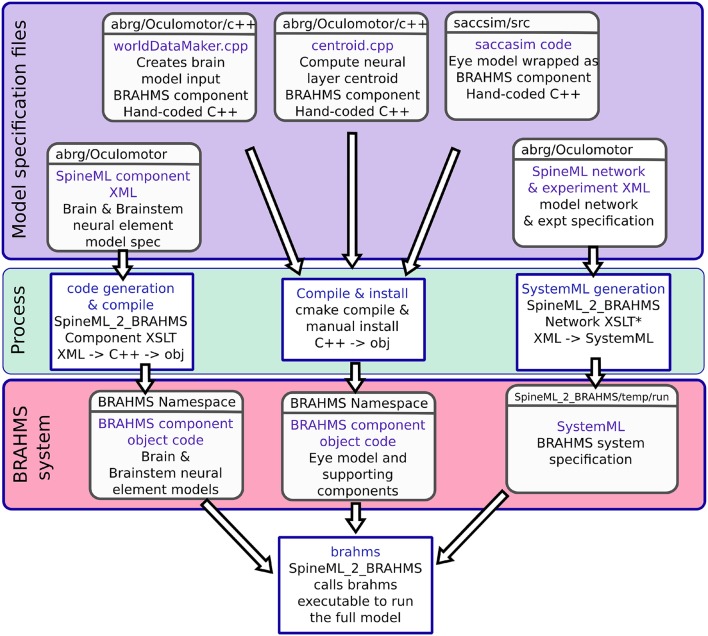
The model framework. **(A)** The model is specified using a combination of declarative XML files and hand-coded C++. These original model specifications are shown within the blue box. **(B)** The green box shows the processes which are applied to the model specification to produce the BRAHMS system. Most of the process is defined within the scripts which make up SpineML_2_BRAHMS, but the hand-written components must be manually compiled and installed within the BRAHMS Namespace, allowing the BRAHMS executable to locate them at runtime. **(C)** The red box shows the resulting BRAHMS system ready to be executed by the BRAHMS executable. In practice, this call is made by SpineML_2_BRAHMS.

In addition to the brain model components, all of which are code-generated using SpineML_2_BRAHMS as described above, two hand-written components are integrated into the model: The biomechanical eye model and a sensory input component. The sensory input component takes the eye's rotational state and the state of the experimental luminances and projects a retinotopic activity map into the brain model. Both of these BRAHMS components were hand-written in C++. To incorporate these components into the SpineML model, a SpineML_2_BRAHMS *external.xsl* file was used. The external.xsl file scheme for incorporating external BRAHMS components into a SpineML model was a new SpineML_2_BRAHMS feature motivated by the current work. Figure 6 shows the workflow, in which the model specification files (blue box—a combination of SpineML files and C++ code), are processed (green box) into a BRAHMS system (red box).

### 2.6. Integrating the models and closing the loop

The Cope-Chambers model closes its loop by passing the centroid of activity in SC_deep (once it has surpassed a threshold) back to the code that controls the world, which then uses this location to instantaneously change the model's view of the world. In our extended model, it was necessary to connect the output of the brain model back to its input via the saccadic burst generator model and the biomechanical eye. The resulting state of the eye, rather than the centroid of the superior colliculus, was used to compute the input to the brain, given the luminances visible in the world.

Thus, the information flow in the model is as follows: Luminances in the world have their locations computed in the eye's frame of reference, based on the rotational state of the eye. The locations of the luminances on the retina are transformed into a retinotopic co-ordinate system which determines the activity in the “World” population (named to mean the “world as the brain sees it,” rather than the world frame of reference) which is the input for the brain model. The target luminance for a saccade is selected, as described earlier, via cortical and sub-cortical loops through the basal ganglia model and activity for the winning end-point builds up in the deep layer of superior colliculus. This activity excites the 6 channels of the saccadic burst generator in the correct proportions for the saccade. The motoneurons, which are the output of the SBG, send a rate-code signal (normalized between 0 and 1) into the biomechanical eye model. The rotational state of the eye model is fed back to participate in the computation of the retinotopic luminance activity in “World,” completing the loop.

A number of studies have considered the form of the connection between the deeper layers of the superior colliculus and the saccadic burst generator (Van Gisbergen et al., 1985; Ottes et al., 1986; Waitzman et al., 1991; Arai et al., 1994; Groh, 2001; Goossens, 2006; Tabareau et al., 2007; van Opstal and Goossens, 2008; Goossens and van Opstal, 2012), which has become known as the spatial temporal transform (STT). The spatial aspect of the transform is thought to be implemented by a weight-mapping (Arai et al., 1994; Tabareau et al., 2007). Although there is no definitive experimental proof for such a mapping, there exists evidence for spatially variable synapse density (Herrero et al., 1998; Moschovakis et al., 1998) and connection density (Grantyn et al., 2002) and we therefore adopt the idea. Arai and co-workers trained a 20 by 20 neural network model of the superior colliculus to discover the weight map under the assumption of 2D Gaussian activation profiles (Arai et al., 1994)—that is, they assumed that the activity in superior colliculus for any saccade was a size-invariant 2D Gaussian hill of activity. The training approach of Arai et al. (1994) was not feasible in this study due to the length of time required to run our model and its stochasticity, which meant multiple runs of the model were necessary in order to generate output statistics. map, obtained by inverting the mapping of Ottes et al. (1986) and the assumption of invariant 2D Gaussian activity profiles in SC, which is equivalent to:

(24) $$w(r,\phi )=i\;e^{jr}\;\sin\left(l\phi +k\right)$$

where *r* and ϕ are co-ordinates on the collicular map and *i*, *j*, *k* and *l* are parameters of the function (compare with Equation 3 of Tabareau et al., 2007). As they found it closely resembles the results of Arai et al. (1994), and it is a simple formulation, we considered it as the means to generate the six weight maps in our own model. One barrier to the use of this weight map was the Cope-Chambers model's violation of the *invariant integral hypothesis*. This states that the number of spikes emitted by a neural element during a saccade (or in our model, the integral of the neuron's output during the saccade) should be a function only of its position within the hill of collicular activity. That is, for any time-dependent hill of activity A(z,t) at **z** = (*r*, ϕ) on the collicular surface, the integrated activity *A*_**x**_ in an element at a vector **x** away from **z** is

(25) $$A_{x}=\int_{t}A(z-x,t)\;dt$$

which is invariant for all **z**. This requirement is fulfilled by spatially invariant 2D Gaussian profiles, whose time-course (how quickly they grow and then diminish) is always the same.

However, the very mapping on which the Tabareau et al. (2007) result is based leads to a very *variant* activity profile in the SC_deep layer of the Cope-Chambers model. A luminance of a given size which excites activity near to the fovea causes activity in a large number of neurons in each retinotopic layer, whereas activity far from the fovea excites a much smaller region. This effect is clearly demonstrated in Figure 3 for equal sized targets both on and distal from the fovea.

**Figure 7:**
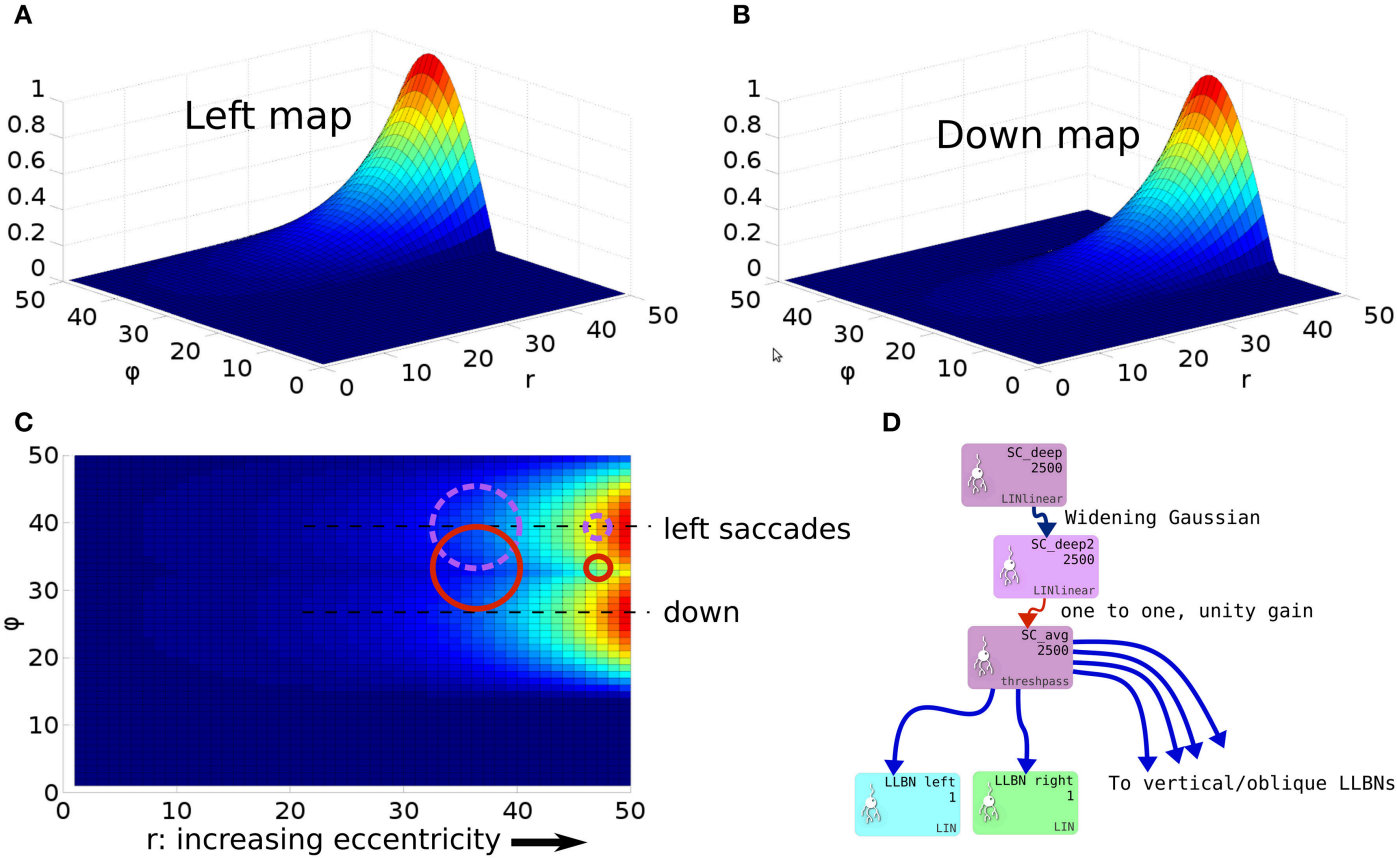
**(A,B)** Tabareau-style weight maps for “left” and “down” components of a saccade. **(C)** The two weight maps in **(A,B)** shown on the same graph, viewed from above. Circles show the locations of potential hills of activity. Purple dashed circles encode saccades left; red circles encode saccades with both a left and a down component. **(D)** Showing the additional deep layer of superior colliculus (SC_deep2) and the output layer (SC_avg, named for the fact that in an earlier version of the model, it received the output of the centroid of SC_deep). The widening Gaussian projection is shown as the arrow between SC_deep and SC_deep2.

To understand the need for this invariance, consider the effect of a 2D Gaussian hill in SC_deep which elicits a successful horizontal saccade of 10° using the weight maps shown in Figures 7A–C. Activity from the 2D Gaussian (schematically represented as the large purple dashed circle in Figure 7C), passing through the weight maps will excite the superior and inferior rectus channels by an equal, balanced amount, so these cancel out, allowing the eye movement to be horizontal. The amount of activation passed to the lateral rectus muscle results from a convolution of the Gaussian and the exponential component of the weight map relationship in Equation (24). If the Gaussian hill now appears further along the collicular surface, coding for a 20° saccade, *and also becomes smaller* (small purple dashed circle), we can still argue that the vertical component signals to superior/inferior rectus muscles will cancel out, and we could imagine that the exponential component of Equation (24) is correctly parameterized to compensate for the smaller hill. Now consider a 2-D Gaussian hill which codes for a 10° saccade which is “down, and to the right” in equal proportions (large red circle). That means that the hill will sit on the boundary between the weight maps for the “down,” and the “left” muscles. Now, if the hill moves to the *r* = 20° location on the colliculus (small red circle), *and also reduces in size*, it will excite only the periphery of the sine; the exponential increase of the map along *r* is not guaranteed to compensate for the reduction in the convolution of Gaussian hill and the sinusoidally varying component of the weight map along the ϕ axis in Figure 7A.

This led us to hypothesize that the retinotopic mapping to the SBG be preceded by an associated widening projection field such that the hill of activity in a “final” deep layer of superior colliculus is invariant with position on the map. There are a number of locations in the system in which this widening projection field could exist. It could be implemented in the projections between the retinal populations and the superficial layer of SC along with the projection between the World and the FEF population. However, this would affect activity within the basal ganglia of the model, contradicting a result in Cope et al. (2017) which explains the “hockey stick” profile for saccade latency as a function of saccade eccentricity. Instead, we suggest that a widening projection field is encoded within the superior colliculus itself, a complex, multi-layered structure which could quite plausibly support such a function. Indeed, such widening activity can be seen in the stimulation experiments in Vokoun et al. (2010) and Vokoun et al. (2014). In Ghitani et al. (2014), from the same research group, evidence is presented for an excitatory and widely projecting pathway from the stratum griseum intermediale (equivalent to our SC_deep) to the more superficial layers stratum opticum and stratum griseum superficiale. Although this pathway is a “wide” projecting field, the experiments do not indicate whether the projection *widens* along the rostral-caudal axis of the SC. Bayguinov et al. (2015) present evidence for another projecting field within SC whose connectivity pattern *does* change along the rostral-caudal axis. This projection is inhibitory in nature. Although neither of these results precisely match the widening, excitatory projection field hypothesized here, they do indicate that such connection patterns are plausible. Although in this work we do not model the SC in detail, we extended the model with a third functional layer named SC_deep2, shown in Figure 7D (Cope-Chambers has only the two layers SC_sup and SC_deep). We introduced a widening projection based on a Gaussian projection field whose width, σ(*r*) varies in inverse proportion to the magnification factor, *M*(*r*), given in Equation (14) according to:

(26) $$\sigma (r)=\frac{m_{\sigma }}{M(r)}-\frac{m_{\sigma }}{M^{0}}+\sigma_{0}\;r>r_{0}$$

*m*_σ_ is a scalar parameter which determines the “magnitude of the widening.” *M*^0^ is the “starting” magnification factor. Within the foveal region (0 ≤ *r* ≤ *r*_0_), the projection field is not allowed to widen and so

(27) $$\sigma (r)=\sigma_{0}\;r\leq r_{0}$$

which makes σ_0_ the width of the Gaussian projection field within the foveal region. (Note that the value chosen for the width of the foveal region, *r*_0_ is not identical to the foveal shift parameter used in the *DecayingAtFovea* projections into striatum.) The *Widening Gaussian* projection weight, *w*(*r, d*) is then computed as:

(28) $$w(r,d)=e^{-\frac{d^{2}}{2\sigma {\left(r\right)}^{2}}}$$

where *d* is the distance between the source and destination elements in the collicular plane. *m*_σ_ was set to 50, σ_0_ was 0.3, *M*^0^ was 12.43 and *r*_0_ was 20.

A further issue regarding the use of the theoretical weight map in Tabareau et al. (2007) was that it does not consider the existence of the oblique extraocular muscles. There is evidence that only two dimensional information is encoded in superior colliculus (Wurtz and Goldberg, 1972; Van Opstal et al., 1991; Hepp et al., 1993), but the eye is actuated by six extraocular muscles. In order to find out a possible form for the input to the oblique muscles we carried out a training process which depended on a centroid computation in SC_deep and was designed to maintain a null torsional eye rotation for all saccade end-points. For the four rectus muscles, the resulting weight map solutions resembled those found by Arai et al. (1994). The trained maps for the oblique muscles had a form very close to those for the inferior and superior rectus channels, but with a smaller magnitude. The inferior oblique map resembled the superior rectus map and the superior oblique map resembled the inferior rectus. When parameterizing the theoretical weight maps, we set the inferior/superior oblique maps to be 1/10th of the superior/inferior rectus maps, respectively. Interestingly, this suggests that there is a built-in synergy between the vertical and oblique channels in the eye, although the results will show there is some systematic change in the oblique error with saccade end-point location.

Tabareau et al. (2007) give a formulation for the weight maps in which it is possible to project both a positive and a negative weight. In our model, all projections from SC_deep are excitatory. This means that each channel has a weight which follows the form:

(29) $$w(r,\phi )=i\;e^{jr}\;\sin\left(\frac{2\pi \phi }{W_{nfs}}+k\right)$$

where *i*, *j* and *k* are per-channel parameters for the weight maps. *k* is determined by the mapping. Only the positive part of the sine is utilized. *i* and *j* are parameters to be found.

The saccadic burst generator model was originally conceived with the assumption of a step input, which returns to zero activity at a suitable time to curtail the saccade and avoid staircase saccades (Gancarz and Grossberg, 1998). In our model there is no such mechanism to reduce activity in SC_deep, and elsewhere. Although a successful, accurate saccade toward a target luminance will remove the excitation which caused the activity in SC_deep by bringing the target luminance within the masked, foveal region, the activity in SC decays too slowly to avoid additional saccadic movements. We found it necessary to hypothesize an inhibitory feedback mechanism from the SBG to the brain model. This is shown in Figure 4, which indicates how the output from the inhibitory burst neurons (IBN) of the SBG model are used to feed back an inhibitory signal to the SC_deep, thalamus and FEF populations in the brain model, resetting them ready for the next saccade. There is evidence for inhibitory projections to SC from the propositus hypoglossi nucleus (Corvisier and Hardy, 1991), which lies within the brainstem, upstream from motoneurons, and has been shown to encode eye velocity (Dale and Cullen, 2013).

The output signals from the six channels of the SBG were connected to the six motoneuron inputs of the biomechanical eye. The signal was normalized; a value of 1 meaning that all the motoneurons in the output population were firing at their maximum rate and the force exerted by the relevant extraocular muscle was maximal. Channels innervated extraocular muscles as follows: Up: superior rectus; Down: inferior rectus; Right: medial rectus; Left: lateral rectus; Z+: superior oblique; Z-: inferior oblique. Because the medial rectus induces a rightward rotation of the eye, our single virtual eye is a *left* eye. The OpenSim implementation of the biomechanical eye was “wrapped” (in the software sense) in a BRAHMS component. This made it possible to integrate the OpenSim model into the BRAHMS framework. The wrapper ensured that the input and output signals were correctly transferred and, importantly, handled the disparity in the solver timesteps used in the OpenSim model (25 ms) and the neural model (1 ms). This was achieved by having the BRAHMS wrapper create a separate thread to run the OpenSim model. The BRAHMS wrapper component was called on each 1 ms timestep, receiving the instantaneous activations from the motoneurons in the SBG. These activations, and the current simulation time, were written into a shared memory area, accessible by the OpenSim thread. Running independently, the OpenSim thread would update its inputs (using the most recent values in the shared memory area) whenever the simulation time had increased by 25 ms. It would then recompute its outputs (the rotational state of the eye) and write these into the same shared memory. The BRAHMS wrapper would update its outputs whenever they were changed in the shared memory by the OpenSim thread. A direct connection of the six outputs of the BRAHMS eye model component to the six inputs of the worldDataMaker BRAHMS component was specified in the SpineML_2_BRAHMS external.xsl file.

The eye model outputs its rotational state at each timestep. The rotational state is used to compute the view of the world in the eye's frame of reference. To simplify the calculation, the luminances exist on a spherical surface at the center of which is the eye. A hand-coded BRAHMS component called worldDataMaker computes the projection of the luminances into the eye's frame of reference and then converts this representation into a retinotopic map to pass into the brain model. The input to the brain model is thus able to change continuously, on every timestep, rather than in a step-wise fashion when a saccade occurs, as in the Cope-Chambers model.

In the worldDataMaker BRAHMS component, the rotational state of the eye was used to construct Euler rotation matrices which transformed between the world's frame of reference and the eye's frame of reference. The worldDataMaker component received a specification of the world luminances in a JSON file called luminances.json at the start of each simulation. This file specified the position, shape, size, luminance, appearance time and disappearance time of an arbitrary number of luminances. With this information, the instantaneous rotational state of the eye and the parameters of the retinotopic transform, it was able to compute the instantaneous input to the brain model.

The final models, on which the results of this paper are based are named “TModel3,” “TModel4” and “TModel5.” Descriptions of these, and earlier versions of the model can be found in the code repository given in Supplemental Data.

## 3. Results

### 3.1. Weight maps

**Figure 8:**
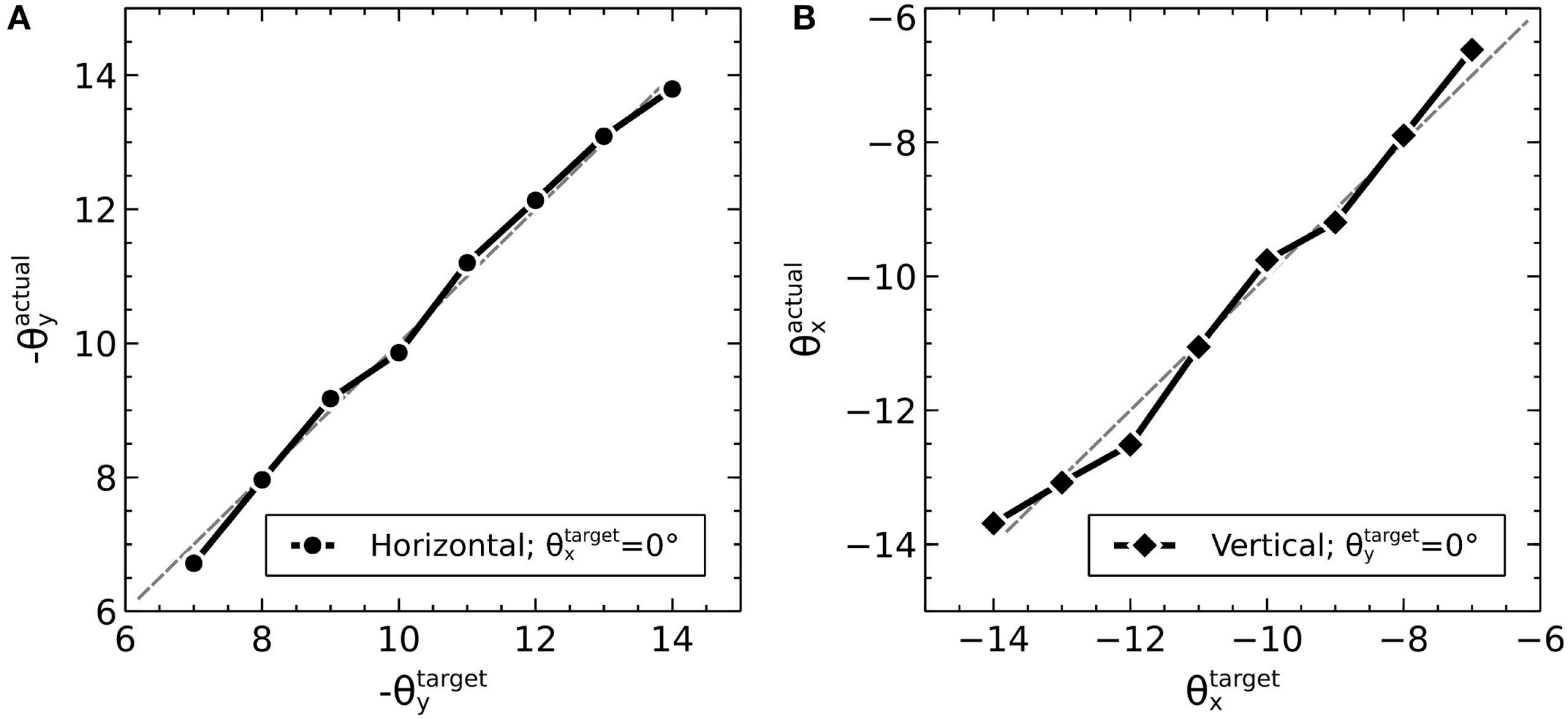
Accuracy of single saccades at different target eccentricities for fixation luminance 0.2 and target luminance 0.3. **(A)** Horizontal rotations about the y axis. **(B)** Vertical rotations of the eye about the x-axis.

We found the best parameters for the exponential in Equation (29) (*i* and *j*) by a manual tuning process. After selecting values for *i* and *j* in either the horizontal or vertical/oblique channels, we ran the model 6 times at each of 8 target eccentricities (7°–14°) which were purely in the direction of the newly parameterized channel. The training saccades were produced as described below in section 3.3, with the same fixation and target luminances (crosses of magnitude 0.2 and 0.3) but with the fixation offset and target onset occurring at 0.2 s. We measured the end-point of the saccade by detecting the location at which the saccade velocity had dropped below 0.005 of its peak. We iterated until the mean saccade endpoint plotted vs. target was close to the ideal straight line—see Figures 8A,B. We applied the same parameters to both directions of each channel; *i*_*up*_ = *i*_*down*_ = 0.00195, *j*_*up*_ = *j*_*down*_ = 0.075, *i*_*left*_ = *i*_*right*_ = 0.0016 and *j*_*left*_ = *j*_*right*_ = 0.067.

**Figure 9:**
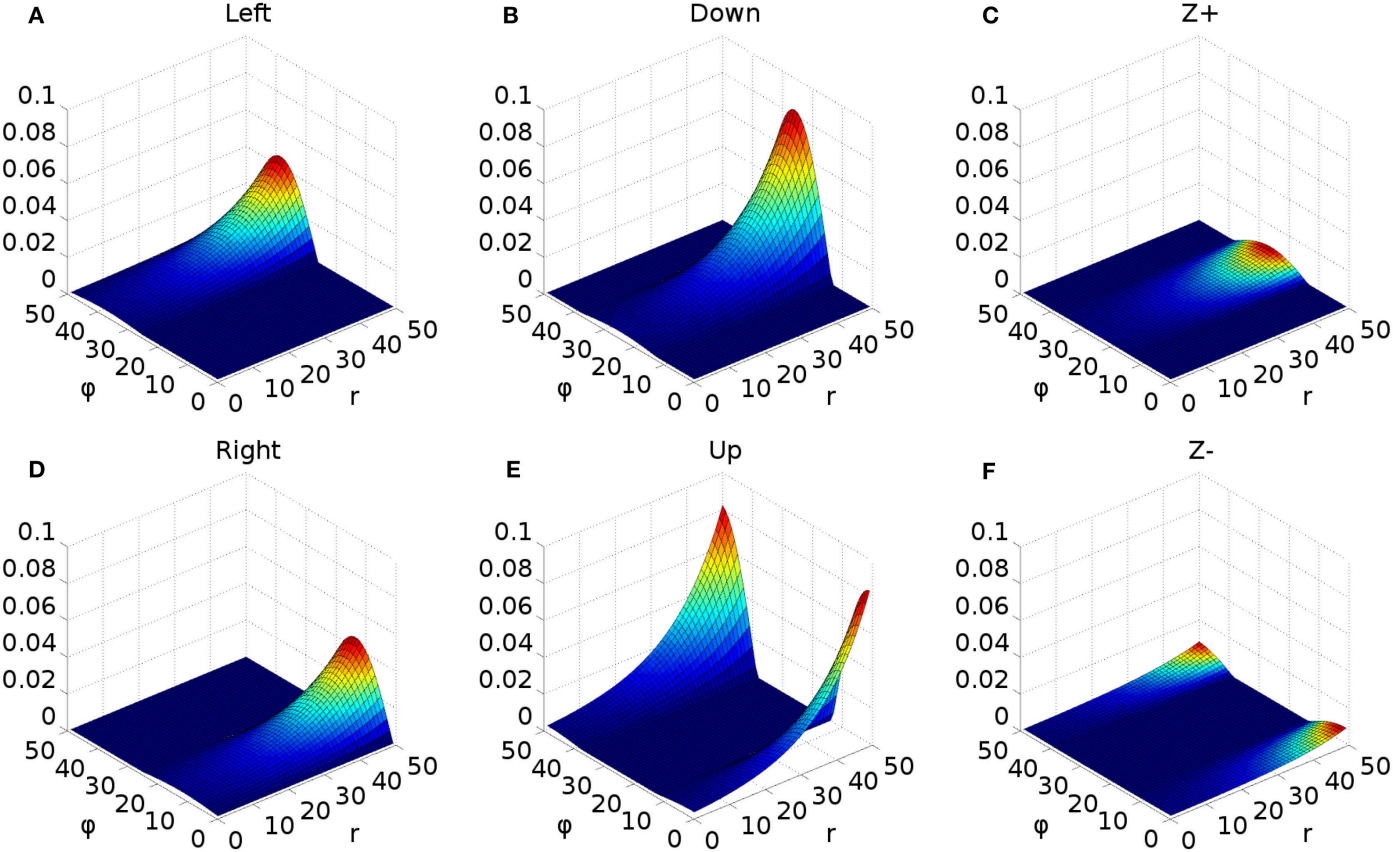
Weight maps for the connections between the output layer of superior colliculus and the six long lead burst neurons of the saccadic burst generator model. Each map increases exponentially with increasing *r*, multiplied by cosine(ϕ) about its “active” axis. **(A)** Weight map for leftward movements which innervates the lateral rectus in this single left-eye model. **(B)** Map for downward movements; innervates the inferior rectus. **(C)** Superior oblique muscle weight map. **(D)** Weight map for rightward movements/medial rectus muscle. **(E)** Weight map for the superior rectus muscle which generates upward movements. **(F)** Inferior oblique map.

The resulting weight maps (where the oblique maps are 1/10th of the vertical maps, as described earlier) are shown in Figure 9. First, recall that the *r* axis of the neural surface corresponds to the amplitude of a saccade and the ϕ axis indicates the polar direction of the saccade, as described in section 2.2.2 and Figure 3. Figure 9A shows the weight map for the muscle which rotates the eye to the left. As we modeled a left eye, this actuates the lateral rectus muscle. The exponential rise of Equation (29) (for experimental evidence, see Figures 7, 8 of Herrero et al., 1998) is seen in the *r* direction; as *r* increases, so the connection strength to the SBG channel rises exponentially. The connection strength is greatest along the center line, for a value of ϕ which corresponds to a purely leftward movement. Note that ϕ is presented in neural co-ordinates, and not in degrees or radians; 1 ≤ ϕ ≤ 50 corresponds to a range of 0 to 360°; ϕ = 38.5 corresponds to movements left. The connection strength drops away sinusoidally as ϕ moves away from the center line at ϕ = 38.5. In regions of the map for which there is no leftward movement, that is, in the half of the map which corresponds to any movement with a rightward component, the “left” weight map is 0. Figure 9D shows the weight map for rightward movements, actuating the medial rectus muscle of the eye. The line of maximum connection strength is along ϕ = 13.5. The map is a mirror of Figure 9A, reflected about the line ϕ = 26. The “left” and “right” weight maps are orthogonal; the non-zero region of the “left” map is zero in the “right” map and vice versa. Figures 9B,D show the weight maps for downward and upward eye movements; the “down” map activates the SBG channel for the inferior rectus muscle, the “up” map activates the superior rectus. Note that “down” is not orthogonal either to “left” or “right” because a saccade down and left is achieved by simultaneously activating both the lateral and inferior rectus muscles. However, the “up” map *is* orthogonal to the “down” map and spans the edges of the surface where ϕ rolls over from 1 to 50. The line of maximum connection strength for the “up” map is along ϕ = 1; for “down” ϕ = 26. Based on the training described in section 2.6, the maps driving the superior oblique (“Z+”) and inferior oblique (“Z−”) muscles were set to 1/10^th^ of the “down” and “up” maps.

### 3.2. Saccade accuracy

**Figure 10:**
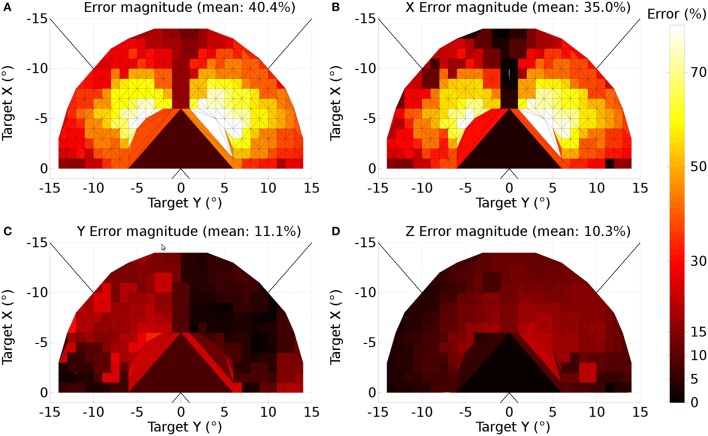
The end-point error surface for the original, naïve model (TModel3). **(A)** The ratio of the magnitudes of the total error vector and the target vector, expressed as a percentage. **(B)** The ratio of the magnitude of the *x* component of the error vector to the magnitude of the target vector, expressed as a percentage. **(C)** As **(B)** but for the *y* component. **(D)** As **(B)**, for *z* component. All color maps are shown with the same scale. The target rotations, $\theta_{x}^{t}$ and $\theta_{y}^{t}$ are denoted “Target X” and “Target Y” in the figure.

In Figure 8, we showed the result of running the model to targets located on the principle axes, on which the model was trained. We then simulated single saccades to targets in one hemifield of the eye's field of view, with eccentricities between 6 and 14.5°. As in the training, we ran the simulation 6 times for each target, $\theta^{t}=\left(\theta_{x}^{t},\theta_{y}^{t},0\right)$ to obtain mean saccade end-points. Figure 10 shows saccade accuracy results for an entire hemifield in the naïve model which passed the output of SC_deep directly to SBG via the weight maps. The ratio of the magnitude of the error vector to the magnitude of the target vector is plotted using a color map. This ratio is shown for the full, three dimensional error vector in Figure 10A and for the *x*, *y* and *z* components in Figures 10B,C. Inspection of Figure 10A shows that the end-point error is minimal along the principle axes ($\theta_{x}^{t}=$0 or $\theta_{y}^{t}=$0) and maximal near the 45° oblique targets (blue lines) with the end point error as high as 80% of the programmed saccade magnitude. The *x* component error map in Figure 10B shows the same trend, mirrored about the “Target X” axis, whereas the *y* and *z* component errors are, relatively, much smaller. Because the *x* component of the error is clearly contributing to end point errors which would not be considered “on target,” especially for oblique saccades, we considered the effect of the non-uniform size of the hill of activity in SC_deep.

In our model, the location, *size* and shape of activity in FEF, the basal ganglia, thalamus and superior colliculus is eccentricity dependent, in line with the retinotopic mapping stated by Ottes et al. (1986). More eccentric targets generate reduced activity, because fewer retinal neurons are excited far from the fovea. Cope et al. (2017) showed that this relationship can explain increased saccadic latencies for distal targets, resulting from reduced activity in the decision making circuitry of the basal ganglia. However, the notion that activity in superior colliculus is eccentricity-dependent conflicts with the result of Tabareau et al. (2007), who showed that an invariant hill of activity was required if this complex logarithmic weight mapping was to be used to drive a two-degree-of-freedom saccadic burst generator, and also with experimental findings, which do not show significant eccentricity dependence, at least in the burst layer (Anderson et al., 1998).

To bring our model in line with these results, whilst maintaining the eccentricity dependent activity in basal ganglia, we hypothesized that a “widening projection” exists between two maps in superior colliculus. As described in section 2.6, there is now experimental evidence for similar projections (Ghitani et al., 2014; Bayguinov et al., 2015) making this a plausible suggestion. Activities in one SC_deep layer remains eccentricity-dependent, with loops back to thalamus and cortex and through basal ganglia. This activity is then fed through a projection, which applies a Gaussian projection field, whose width increases with increasing stimulus eccentricity according to Equation (28). The activity in this second SC_deep layer is then fed to the weight maps of the SBG. This model was called “TModel4.” TModel4 was parameterized such that its horizontal and vertical error was similar—so that its equivalent of Figure 8 showed a similar sum of squares error.

**Figure 11:**
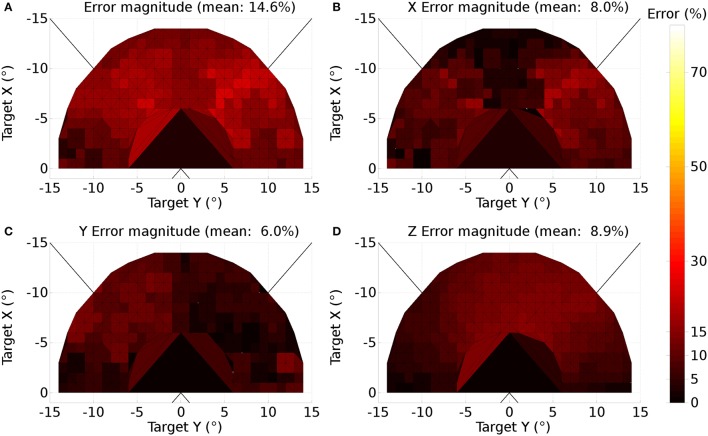
The end-point error surface for the model in which a widening projection field was added to the model of the superior colliculus. **(A)** The ratio of the magnitudes of the total error vector and the target vector, expressed as a percentage. **(B)** The ratio of the magnitude of the *x* component of the error vector to the magnitude of the target vector, expressed as a percentage. **(C)** As **(B)** but for the *y* component. **(D)** As **(B)**, for *z* component. All color maps are shown with the same scale. The target rotations, $\theta_{x}^{t}$ and $\theta_{y}^{t}$ are denoted “Target X” and “Target Y” in the figure. Note that the range of the color scale is 0 to 20%, a much smaller range than the range in Figure 10.

Figures 11A–D show the percentage errors for TModel4. First of all, note that the error magnitudes are much smaller. The mean errors are smaller for every axis. The largest errors produced by the model are approximately 15%, which are within the boundaries of what some authors regard as an accurate saccade (McPeek and Keller, 2002; McPeek, 2006). The magnitude of the largest error vector is ~ 1.5°.

This result indicates that the exponential part of the Ottes et al. weight map from SC to the SBG cannot on its own compensate for the eccentricity-dependent size of the hill of activity. The introduction of a widening projection field substantially improves the mean accuracy of saccades across the field of view. We therefore suggest that the transformation between retinotopically mapped activity, and eccentricity-independent activity width occurs within the superior colliculus and works alongside a simple, monotonically increasing weight map between SC and the SBG channels.

### 3.3. Single saccades

**Figure 12:**
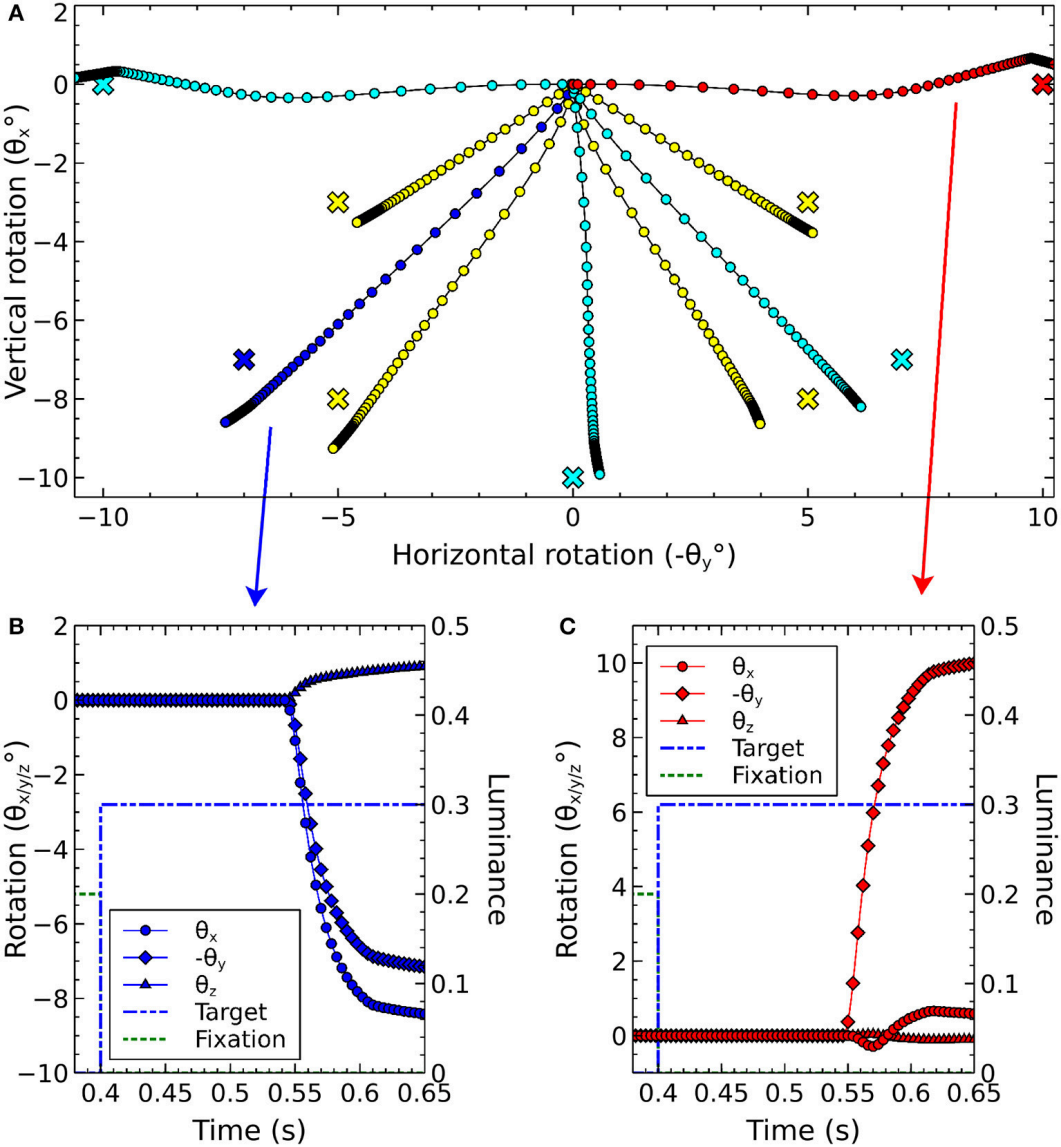
Representative single saccades. **(A)** Trajectories from 9 saccades to a single target at 9 different locations. In each case, a fixation cross luminance of magnitude 0.2 was displayed at (0,0), the start position of the eye, until time 0.4 s. The target luminance, magnitude 0.3, was illuminated at time 0.4 s. Trajectory shape is dependent on the target position, and there is a variable amount of error in the end-points achieved by the model. Color is used in this diagram as an aid to distinguishing different saccades and their targets; for a given saccade, the target location is given by the cross of the same color closest to the end of the trajectory. **(B)** The three rotational components of the “dark blue” saccade, to target location (−7, −7). **(C)** The three rotational components of the “red” saccade, to target location (0, −10).

Having finalized the model by setting the weight maps, we then proceeded to exercise the model (TModel4), starting with saccades to a single target; prosaccades. Figure 12A shows 9 representative saccades to a single target luminance. Initially, the eye had rotational state θ_*x*_ = θ_*y*_ = θ_*z*_ = 0 with its fovea directed at a fixation luminance cross (span 6°, bar width 2°) of magnitude 0.2 (in arbitrary units). At a simulation time of 0.4 s, the fixation luminance was set to 0 and a target luminance cross of the same dimensions as the fixation but with magnitude 0.3 was illuminated at one of the 9 different locations, marked by crosses in Figure 12A. The resulting trajectories are plotted, with color indicating the relationship between trajectories and target crosses. The approximate end-point error is visible in this figure, although the last point in each trajectory is the saccade position at 0.8 s and not the velocity-based end-point described above. Figures 12B,C show the rotational components of the blue and red trajectories in Figure 12A along with the target and fixation luminance values. Rotations are the eye's Euler rotational components in the world frame of reference.

### 3.4. Saccade latencies

**Figure 13:**
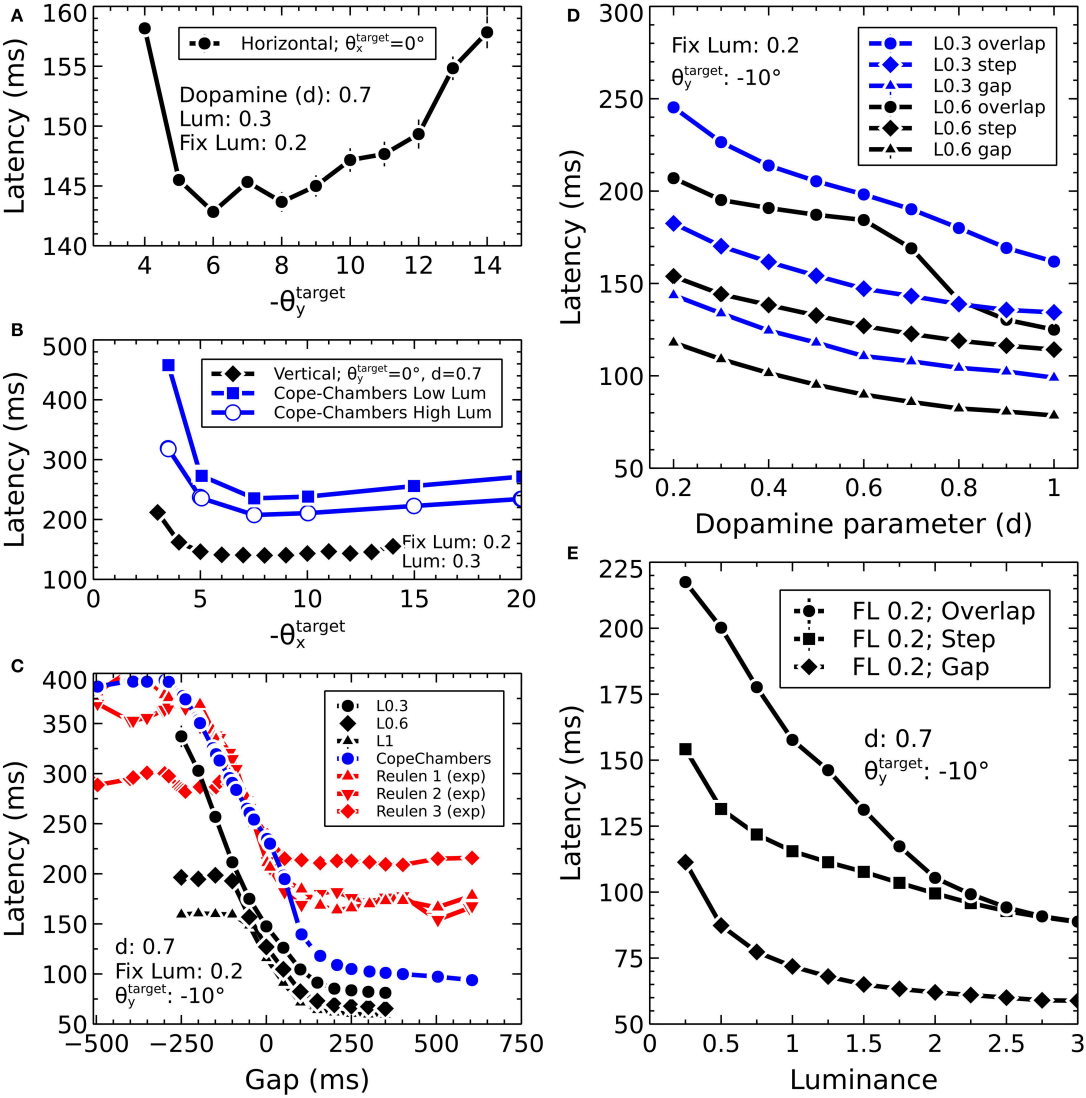
Exploring saccade latencies. **(A)** Latency to first movement as a function of target eccentricity for horizontal targets. **(B)** Latency vs. eccentricity for vertical targets. **(C)** Latency vs. gap at three different luminance values. The data are shown alongside the Cope-Chambers model results from Figure 5 of Cope et al. (2017) in blue, and the experimental results used in that model in red. The fixation luminance for the Cope-Chamberscurve was 0.5, the target luminance 0.6 and the target eccentricity was 8°. The difference between the Cope-Chambers model data and the data from the current model results from the different mechanism by which activity in SC_deep causes a movement, the differing target angle and the reduced fixation luminance used here. **(D)** The effect of the dopamine parameter on saccade latencies in gap, step and overlap conditions, for two different target luminances. **(E)** Saccade vs. luminance showing gradual transition between reflexive and express behavior.

To verify that our implementation of the brain model has the same functionality as that reported in Cope et al. (2017), we investigated the effect on saccadic response times of: target eccentricity; and any gap or overlap between fixation off-time and target on-time. We showed that the full model reproduces the “hockey stick” shape shown in Figure 7 of Cope et al. (2017) and discovered in experimental data (Reulen, 1984) for horizontal (Figure 13A), vertical (Figure 13B) and oblique saccades (not shown). The latency increases with eccentricity far from the fovea because the retinotopic mapping reduces the activity in the basal ganglia for more eccentric targets (see Figure 3). Closer to the fovea, the effect of the foveal mask on the activity in FEF again leads to reduced input into the basal ganglia and an increased time to achieve disinhibition in SNr.

Figure 13C shows latencies achieved when varying the time between fixation offset and target onset. This is termed the *gap condition*; and is represented by a scalar value which, if positive, refers to a gap between fixation offset and target onset, and when negative, signifies an overlap, with the fixation luminance persisting past the time at which the target is illuminated. A negative gap is also termed an *overlap*. Again, we verify the behavior presented in Cope et al. (2017), explained as resulting from the inhibition of the cortico-thalamic loop by SNr. In the gap condition, when the fixation luminance is removed, activity in STN immediately begins to decay, allowing SNr activity to reduce and thereby reducing inhibition on thalamus, allowing the target luminance to build up quickly in FEF, thalamus and through the basal ganglia's striatum and SNr. The shape of the curves in Figure 13C matches the results in Cope et al. (2017) for target luminances of 1 and 0.6; for overlaps longer than 100 ms (gap < −100 ms), the latency becomes constant; the saccade is programmed whilst the fixation is present, with the target luminance inducing sufficient activity in striatum to “break through” the SNr inhibition caused by the fixation. If the target luminance is reduced to 0.3, the balance is altered in favor of the fixation and the latency vs. gap becomes approximately linear and equal to the overlap time plus around 100 ms.

Figure 13D shows the effect of the dopamine parameter on saccade latencies in gap, step and overlap conditions. In general, the effect of decreasing the dopamine parameter was a smooth, monotonic and undramatic increase in saccade latency. However, the data for the overlap condition with a target luminance which was 3 times as bright as the fixation luminance was more interesting. Here we see a transition around a dopamine value if 0.7. Below this value, the basal ganglia is not able to select the target luminance until the fixation is removed, reducing the excitatory drive from STN to SNr, and consequently the inhibition from SNr to the thalamo-cortical loop. For the target luminance 0.6, 0.7 dopamine allows the basal ganglia to select sufficiently well so that the target can build up in the thalamo-cortical loop, in spite of the fixation overlap.

The relationship between latency and the target luminance is given in Figure 13E. This shows latency for a 100 ms gap, step and 100 ms overlap conditions for a given fixation luminance of 0.2, and a horizontally located target at $\theta_{y}^{t}=-10^{{}^{\circ}}$. For the gap condition, we see very short latencies for luminances of about 0.75 and above. Finally, the activity driving these express saccades is initiated by high firing rates in the superficial layer of SC (SCs), which then drives activity in thalamus and through the basal ganglia. A gradual transition from express saccades to reflexive saccades is observed as the contribution of the SCs becomes weaker and the drive from FEF into the thalamo-cortical loop becomes necessary to elicit a saccade. A similar gradual transition, albeit for higher latencies is seen for the step condition. At higher target luminances, the SCs has a greater effect on the activity in the thalamo-cortical loop. However, the activity in STN caused by the fixation luminance increases the latency at all luminance values compared with the gap condition. The overlap condition leads to increased latencies for luminances below 2.5, but meets the step condition above this value, at which the 0.2 fixation luminance appears to have a negligible effect on the system.

### 3.5. Saccade sequences

We now present results derived from the fully parameterized and integrated model; where we took advantage of the fact that it is a closed loop system. This allowed us to present sequences of target luminances and allow the model to direct its fovea at the most salient target.

#### 3.5.1. Out and return

We investigated the behavior of the model for saccade sequences. In one experiment, we illuminated a fixation cross from 0 s until 0.4 s, followed by a target at (0, −10°) from 0.4 s until 0.8 s. Finally, the fixation was again shown from 0.8 s until the end of the simulation at 2 s. This induced a saccade to a 10° eccentricity, followed by a return saccade back to the null point. We noticed some irregularities in the return saccades, which were accurate, but had a significant overshoot. More perplexingly, if the target was switched repeatedly between 0° and 10°, second and subsequent *outward* saccades also showed this overshoot. We found that the cause of these irregularties was the lack (in “TModel4”) of any mechanism to reset the tonic neurons in the SBG after the first saccade. This resulted in TN activity in the left channel *and also* in the right channel. Interestingly, this ensured that, at least for a few, consecutive out-and-return saccades, the saccade accuracy was accidently relatively good, with trajectories resembling experimental data (Bahill and Stark, 1979, p. 6). Had the return saccades not been so accurate, we may have noticed the lack of a tonic neuron reset mechanism and corrected this oversight earlier. Such a mechanism is indeed proposed and included in the connectivity of the (Gancarz and Grossberg, 1998) model. We implemented this feature by adding an additional inhibitory input to the “integrator” SBG component of TModel4, driven by the contralateral EBN population, naming the new model “TModel5.” Now, when the eye is directed toward an eccentric target which is then exchanged with a target at the null point, the EBN activity toward the null point will tend to extinguish the TN activity which was holding the eye at the eccentric position. We verified that none of the single saccade results were affected by this modification.

**Figure 14:**
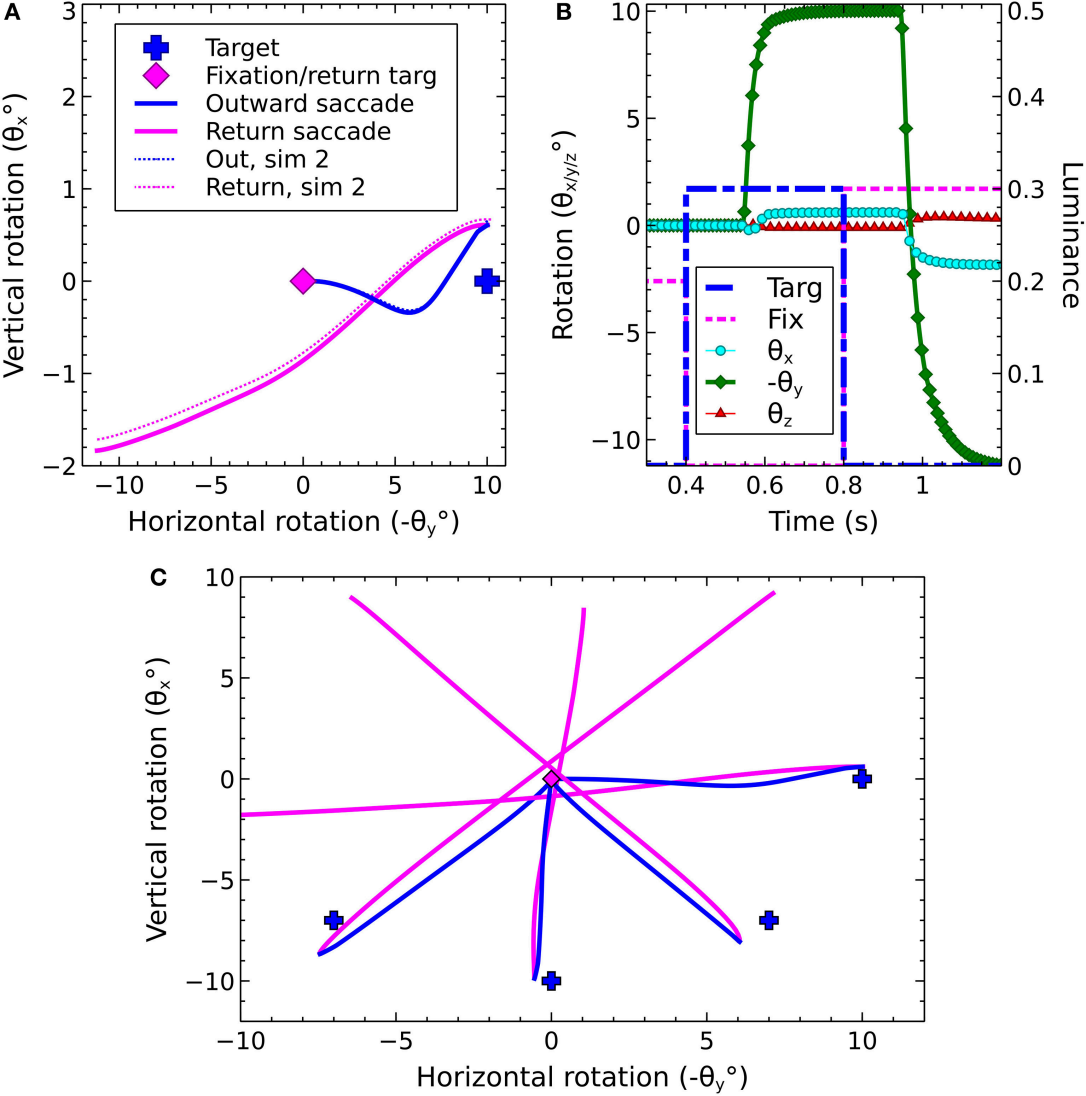
There and back—a saccade to a target, followed by return to the original fixation. **(A)** Out and return saccade to a target at (0,−10°). The outward trajectory is shown in blue, the return in pink. **(B)** Rotational components of the saccade shown in **(A)**. **(C)** Outward and return trajectories for the saccade shown in **(A)** alongside saccades to three other targets.

Figure 14 shows the outward and return trajectories produced by the experiment with the TN reset mechanism. Panel (a) shows the *x* and *y* rotation trajectory; panel (b) shows individual rotational components of the eye. Figure 14C shows out and return trajectories for three other saccade targets; horizontal, vertical and oblique. The trajectories have characteristic shapes and also show some stochastic variation caused by the noise in the model (see dashed trajectories in Figure 14A).

The return trajectories (magenta lines) showed a distinctly different form from the outward trajectories. They overshot their destination (the null point) significantly. This resulted from the removal of the TN activity which was holding the eye at the eccentric target location. Removal of this activity, and thus the static force exerted by the corresponding extraocular muscle, meant that the eye was subject both to a new muscular force toward the null point *alongside* the restorative spring force of the lengthened rectus muscle. This stands as a shortcoming of the model.

#### 3.5.2. Double steps

**Figure 15:**
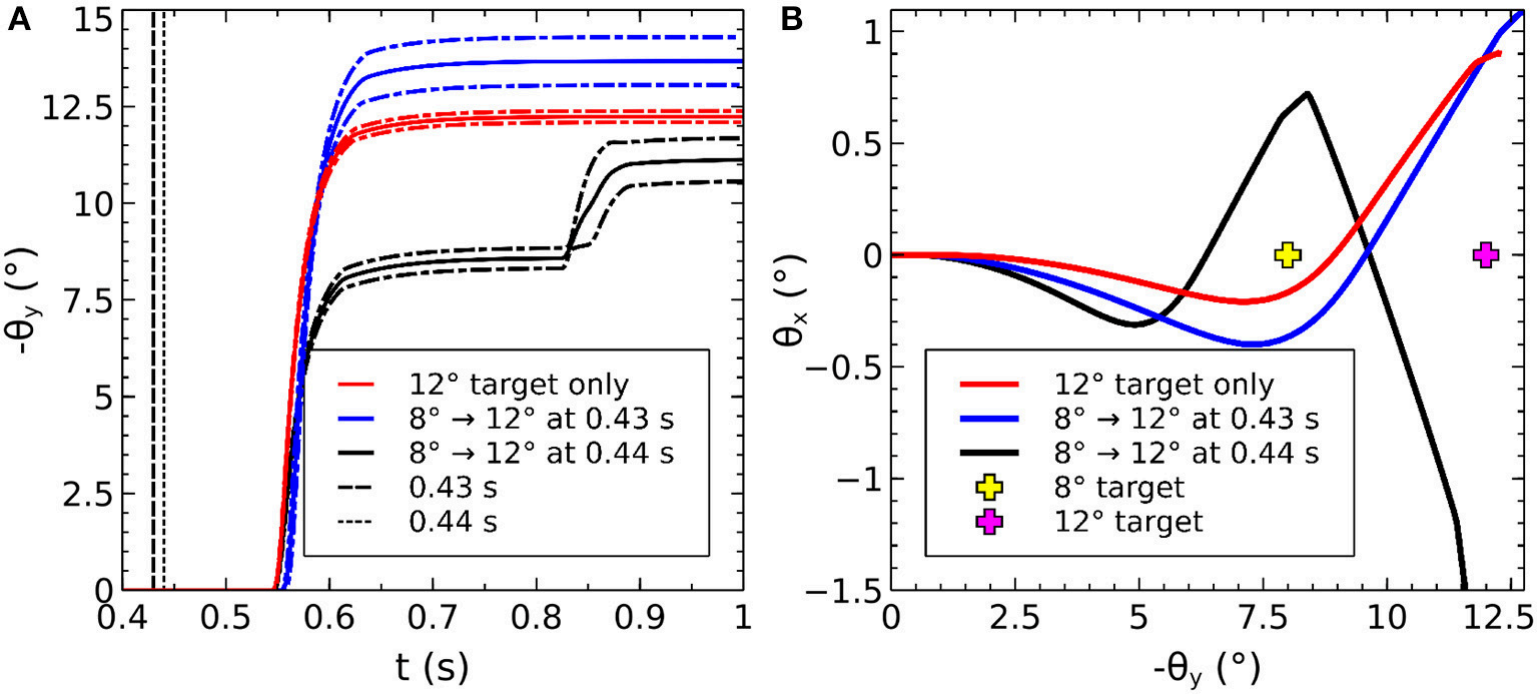
Double steps. The effect of illuminating a first target at 8 or 12°, followed by a second target at 12 or 8°. **(A)** Horizontal rotation of the eye plotted vs. time for a saccade to the 12° target only (red), and to an 8° target at 0.4 s followed by a 12° target after 30 ms (blue) or 40 ms (black). The timings are indicated by vertical lines. When the second target is presented up to 30 ms after the initial target, the initial target has not had time to dominate the output saccade and a saccade to a location close to the second target is made. If the delay is 40 ms or more, the activity from the initial target has time to cause a built up of activity in SC_deep and an initial saccade close to the first target is made, followed, after a longer than usual latency period, with a second saccade closer to the second target. In this graph, the mean of five separate simulations is plotted along with ±1 standard deviation around the mean. **(B)** The θ_*x*_/θ_*y*_ trajectories corresponding to the data presented in **(A)**.

In another experiment, we probed the response of the model to double step stimuli of the type described in Becker and Jürgens (1979). In that work, the response of human subjects was investigated when shown stimuli at 15° and 30° eccentricity with variable delay between the stimuli. If the smaller eccentricity stimulus was shown first, followed by the more distal on the same side of the field of view, this was called a “staircase” presentation. We carried out a “staircase” presentation, shown in Figure 15, where our small eccentricity luminance was at 8° and our more distal luminance was at 12° (both to the right of center). The stimuli could not be presented at 15° and 30° to match the experiment, because 30° saccades were outside the range of the model.

We found that there was a critical time delay between the luminances of about 30 ms. If they were presented with a delay smaller than this value, then a single, slightly hypermetric saccade was made. This response type is called a *final angle response*. A delay greater than 30 ms between the stimuli would lead to double step saccades (a so-called *initial angle response*), with the first saccade arriving at 8° (though with greater variability than normal), and a second saccade being made to a location hypometric of 12° after a pause of about 240 ms. Figure 15A shows the mean trajectories from 5 simulations of the staircase doublestep presentation alongside the result for a single saccade to the final angle of 12°. Dash-dot lines show ±1 standard deviation about the mean. The corresponding trajectories are shown in Figure 15B.

Inspection of the activity maps in FEF and SC_deep (not shown) indicates that when the 8° target is illuminated for 30 ms or more, the activity associated with this target angle is able to dominate the activity, hence the execution of a reasonably accurate saccade. The inhibitory feedback from the SBG then extinguishes activity in FEF, thalamus and SC, which means that a full 200 ms or more is required to allow activity in these populations to build up again in order to make the smaller saccade from 8 to 12°. This is in contrast to experimental findings in which the corrective second saccade is often executed *more quickly* than if it were programmed on its own (Becker and Jürgens, 1979).

## 4. Discussion

The aim of this study was to demonstrate the importance of modeling neurological systems *in concert with* the biomechanical systems with which they have evolved. We hypothesized that by combining existing neurophysiological models with an accurate model of a musculo-skeletal system, and then closing the “agent-environment-agent” loop by allowing the movements of the virtual muscles to modulate sensory feedback to the brain model, shortcomings in the constituent models would be revealed, leading to new knowledge.

To demonstrate the validity of this closed-loop approach, we built an integrated model and then identified the modifications which were necessary to give it the ability to make accurate movements under one type of stimulus. We then examined its behavior with other stimuli. We chose the oculomotor model as a basis for this study because it has only three degrees of freedom, making it one of the simplest musculo-skeletal systems. Furthermore, eye movements fall into several well-defined categories, each being controlled by separate brain circuits, we were therefore justified in modeling a system which produced only saccadic eye movements. Nevertheless, we are aware that we did not create a complete model of the system; no treatment of the cerebellum was attempted, justified because cerebellum appears to have only a minor effect on saccade accuracy (Dean and Porrill, 2008), probably correcting for slow to medium timescale changes in the physical dynamics of the eyeball (Dean et al., 1994).

To summarize our model integration: We combined the Cope-Chambers model (Cope et al., 2017) with a saccadic burst generator model based on the work of Gancarz and Grossberg (1998), using this to drive the input of a new biomechanical eye model. To achieve the spatial transformation from the retinotopic maps of the Cope-Chambers model to the six “muscle channel” inputs for the saccadic burst generator, we used the mapping of Ottes et al. (1986) to produce parameterized weight maps along with an empirically discovered synergy for the torsional weight maps. We introduced an additional transformation to the brain model to achieve invariant sized hills of activity in superior colliculus to fulfill the invariant integral hypothesis of Tabareau et al. (2007). We closed the loop using a software component which transformed a view of a world containing luminous cross shapes into the eye's frame of reference, given its instantaneous rotational state. This component also computed the inverse of the mapping from Ottes et al. (1986) to project the view retinotopically into the brain model. This paper serves to describe how we achieved the integration in order to test our hypothesis, and we intend that the material and methods section, along with the model code itself, will help others to carry out similar studies. We will discuss what can be learned from an integrated model of a combined brain and biomechanical system, using our oculomotor system as an example and then consider how this study compares with other modeling and robotic studies of closed-loop systems.

Our integration approach revealed three ways in which this model fails to provide a full understanding of the saccadic system. In each case, the issue is made clear *as a result of the integration*. This is not to say that other approaches may not also reveal shortcomings; we will see that one of our cases has been independently identified (Groh, 2011).

### 4.1. The need for a widening projection field

The original combination of the Cope-Chambers model with the theoretical weight maps of Ottes et al. (1986) and Tabareau et al. (2007) resulted in a model which was able to produce accurate saccades only along the principle rotational axes (Figure 10). Thus, *the integration of the models* suggested that an additional layer was required to achieve accurate saccades for oblique, as well as for horizontal and vertical saccades. Although the *need* for an invariant integral is discussed in Tabareau et al. (2007) as resulting from their theoretical study, the mechanism by which such an invariant Gaussian hill is generated is not. By combining the models, we were forced to consider this mechanism, and hypothesized that a widening projection field would be a candidate mechanism. The results of Figure 11 indicate that a substantial improvement in accuracy is indeed achieved by this new mechanism.

### 4.2. Saccades from non-null starting positions

The implementation of a biologically accurate model of the eye, and the closed-loop nature of the model makes it very natural to consider how the model will behave when making saccades from arbitrary starting positions, or how it would respond to a sequence of stimuli. This was the motivation for the out-and-return experiment (Figure 14) as well as for the double step experiment (Figure 15). We found that return saccades were substantially affected by the biomechanics of the eye, as the brain and brainstem model had no mechanism to account for the position-dependent restoring forces applied by the eye. This question has been addressed by other authors; Groh (2011) investigates the effect of initial eye position on stimulated saccades and finds a need for the signal in superior colliculus to be modulated by an eye position signal. Ling et al. (2007) shows the existence of a position dependent firing rate offset in abducens neurons. Though we will not speculate here on the mechanism by which return saccades may be made accurate whilst also resetting the activity of tonic neurons in the SBG, it is interesting that in the model in which we omitted to reset TN activity (TModel4), we obtained relatively accurate out-and-return saccades which closely resembled experimental data. We suggest that residual activity in TN populations may offer an explanation for how the restorative force exerted by the elastic oculomotor muscles is compensated for. A comparison of this idea with that of Groh (2011) (that there is a modulation, from a brainstem signal, of the SC readout) would make a subject for a future study. Although these existing studies have highlighted this issue, the inaccurate return saccades which the model makes from eccentric starting positions provide a clear example of the way in which integrating known models into a closed-loop system can highlight deficiencies in the model.

### 4.3. Inhibitory feedback from saccadic burst generator to brain

The third issue raised by the integration of the component models of the saccadic system has, like the return saccades, to do with resetting activity. In this case, rather than the reset of activity in the TN population in the brainstem, it is the question of how the activity in the *brain* model should be reset after each saccade. When a target luminance is projected onto the World population in the model, this induces activity which “reverberates” in loops through FEF, basal ganglia, SC and thalamus. The brainstem contains a mechanism to limit the timescale of a saccade (inhibitory feedback from EBN, via IBN to LLBN; see Figure 4). However, if the activity in SC is not reset, then following the completion of the first saccade, a series of subsequent “staircase” saccades will be executed. There needs to be a mechanism to extinguish activity in SC, but also in FEF and thalamus, as activity in either of these populations can build up and eventually cause repeat activity in SC and another saccade. We added hypothetical inhibitory feedback connections to our model, such that the IBN populations in the SBG would inhibit activity in FEF, thalamus and SC_deep (Figure 4), preventing the occurrence of staircase saccades.

An examination of the behavior of the model when presented with “double-step stimuli” reveals a problem with our inhibitory feedback connections. We found that when double-step stimuli were presented (where an initial target at 8° was replaced with a 12° target after 30 or 40 ms) and a double saccade was made (Figure 15A, black lines) the second saccade latency was *longer* even than the initial saccade. This contrasts with Becker and Jürgens (1979) who find that second, corrective saccades occur with *shorter* latencies. This suggests that the inhibitory reset signal implemented in this model is too strong or has the wrong timescales. This issue highlights the fact that connections *between* component models are quite as important as the connections within each model.

There is some evidence for an inhibitory projection to SC from the brainstem. Corvisier and Hardy (1991) offer evidence for a projection from the propositus hypoglossi nucleus. This lies upstream from motoneurons and (in primates) encodes eye velocity (Dale and Cullen, 2013), rather than head movement velocity. Although the propositus hypoglossi does not lie in exactly the same functional location as our IBN population (instead it sits between TN and MN), it offers a possible inhibitory feedback signal proportional to eye velocity and may help to reduce activity in SC post-saccade. Alternatively, it is possible that activity in FEF and thalamus are reset via a “timed signal.” Feasibly, after activity in FEF exceeds a threshold, an internal, inhibitory feedback signal could be activated. This inhibition should have a timescale of sufficient duration to reduce activity in FEF, thalamus and, via an increase in inhibitory output from SNr, also in SC. Indeed, the cortical microcircuit contains a variety of morphologically distinct GABAergic neurons (Douglas and Martin, 2004) which could fulfill this functionality. A similar mechanism would then be required in SC, to reset activity generated by direct excitation via the retinal-collicular pathway which generates express saccades. Again, SC is a multi-layered structure, containing GABAergic interneurons (Meredith and Ramoa, 1998; Munoz and Istvan, 1998; Helms et al., 2004; Sooksawate et al., 2011) and there is mounting evidence that saccade dynamics are generated within SC (Kaneda et al., 2008; Goossens and van Opstal, 2012; Bayguinov et al., 2015). Thus, a more complex treatment of the SC and FEF regions in the model may well obviate the need for inhibitory feedback from brainstem to SC, FEF and thalamus.

Considering whether a feedback connection, or internal, recurrent inhibition is responsible for activity-reset in the brain model raises a more general question about modeling the central nervous system. We should consider whether inaccuracies within one part of the model may propagate errors through the closed-loop system that cannot be counteracted by another part of the simulation. There is no way to know, from integrating sub-systems, which properties hold true, and which are false. However, by integrating models and examining the behavior of the combined model, we are presented with the right questions to ask of the model and the experimental data. In the case of activity-reset, this is to re-assess whether there exists inhibitory feedback from brainstem to the SC and FEF regions, and to find out how an integrated model with self-regulatory mechanisms in SC and FEF may perform.

The omission of the cerebellum will not have escaped the reader's notice. Whilst many of the nuclei known to be involved in the production of saccadic eye movements are incorporated within the model, the cerebellum is not. The cerebellum is known to play an important rôle in saccade programming (Dean et al., 1994; Schweighofer et al., 1996; Quaia et al., 2000; Kleine, 2003). It may be able to completely replace the functionality of the colliculus when lesioned (Aizawa and Wurtz, 1998; Lefèvre et al., 1998). However, this rôle is typically considered to be one of accuracy tuning (Dean et al., 1994; Barash et al., 1999); operating as an additive model. Furthermore, saccades made by individuals with cerebellar ataxias perform with only moderate loss of saccade accuracy (Barash et al., 1999; Federighi et al., 2011). Because we did not address learning in our model, and because our aim was to demonstrate the utility of integrating brain with biomechanics in order to highlight deficiencies, we considered the omission of the cerebellar nuclei acceptable in the present work.

We have not addressed the question of saccade duration in this paper. Saccade duration is of interest in models which produce two (or three) dimensional saccades, because the dynamics of a saccade follow well known relationships with the saccade eccentricity, regardless of the saccade angle. This causes a problem for models (such as the present one) for which some of the dynamic behavior is generated within orthogonal components. For example, saccade duration increases with target eccentricity. A 10° eccentricity oblique (45° up and right) saccade is composed (approximately) of a 7° upwards component and a 7° rightwards component. If the component based model is responsible for the dynamics, then the 10° oblique saccade would be expected to have the dynamics of a 7° up or 7° right saccade. This is not found in practice, and the components are said to have been stretched, hence the name for this effect “component stretching.” The Gancarz and Grossberg (1998) model is reported to take account of the component stretching effect via the OPN neuron population. We did not find this effect in our implementation of the model; the duration of oblique saccades at a given eccentricity was always substantially different from the duration of the corresponding purely vertical or horizontal saccade. Because there is a somewhat complicated interplay between the dynamics of the superior colliculus driving the dynamic system of the SBG, we feel this is outside the scope of the current work and a subject for a future paper.

### 4.4. Comparison with other studies

We have called this closed-loop, biomimetic modeling approach *computational neurobehavior*, in which a complete, behaving model is constructed, with attention paid to the biological accuracy of each brain and biomechanical sub-system. We are by no means the first researchers to consider this interaction between brain and biomechanics. Integrative approaches to motor control have been referred to as *neuromechanics* by some authors (Nishikawa et al., 2007). This field appears to have developed from detailed and low level studies of the mechanics of muscle control systems, focussing on the neural systems “closest” to the muscle (Chiel et al., 2009). It is evident that a recognition of the importance of sensory input to these systems has evolved within this field. Indeed, Edwards (2010) specifically reviews closed-loop, *neuromechanical simulations* of behavior in three organisms; fly, locust and cat. The term neuromechanical simulation (Pearson et al., 2006; Edwards, 2010) is analogous to our computational neurobehavior, although it does not emphasize the important *behavioral* aspect of the works.

There also exist many closed-loop *robotic* systems which receive sensory input from the world, process that input and generate behavior by activating motor systems (Fend et al., 2004; Yu et al., 2004; Pearson et al., 2007). We now consider whether robotic systems which model biological components *in hardware* could fall within our new category. Using a number of examples, we will attempt to illustrate what we mean by computational neurobehavior. We'll consider which examples fall into the new category and which are covered by other fields of robotics or computational neuroscience.

Pearson et al. (2007) describe a wheeled robot which has a biomimetic whisker sensory system, along with a biomimetic neural system imitating the operation of the rat's sensory processing and controlling the movement of the robot. Fend et al. (2004) is a similar, wheeled, whiskered robot, with a repertoire of three behaviors organized in a subsumption architecture. In both robots, actions that are selected within the brain model drive a non-biologically accurate motor control algorithm to achieve rotational and translational movements. Although both have sensory and processing systems which are guided by biology, the non-biological motor control stage prevents us from considering these as being studies of computational neurobehavior. Instead, we would refer to these as *embodied models*, as described in Bolado-Gomez and Gurney (2013), a study in which the learning behavior of a biomimetic “core” model is embedded within an engineered “architecture” (a wheeled robot) which closes the agent-environment loop. Yu et al. (2004) report on a biomimetic fish robot, whose motor system closely resembles that of the real fish. The robot is able to operate in a closed-loop mode, where sensory input is provided to the non-biomimetic control algorithms from overhead cameras, but its control system is also able to operate in open-loop mode. Neither the control system, nor the sensory system are biologically accurate and we would not describe this study as computational neurobehavior. Nevertheless, the biomechanically accurate motor system they describe has the potential to form part of a computational neurobehavioral study of swimming behavior in the fish, if it were combined in a loop with suitable sensory input and sensory processing models. Knips et al. (2017) is a report of a reach-and-grasp robot arm controlled via a dynamic neural field brain model. The sensory input for this system—its “eyes”—is a Microsoft Kinect sensor; it also has somatosensory feedback from the fingers of the robot's hand. The neural field “brain” controls the seven degrees of freedom of the arm to carry out the reach-and-grasp action. While this robot has closed-loop control and is clearly inspired by biology, it remains a study of robotics and of the improvement of the control of the robot's reach-and-grasp function, rather than a study which aims to learn more about the biology of a primate arm. For this reason, we would describe the study of Knips et al. (2017) as an embodied model.

To summarize, in most closed-loop robotic studies which incorporate neuromimetic models, the hardware forms an “engineered surround architecture” allowing for the examination of the behavior of the embodied model. However, suitably *biomimetic* hardware such as the fish in Yu et al. (2004) would not be excluded from computational neurobehavioral studies, especially if movement is by biomimetic muscle actuators (OHalloran et al., 2008; Wilson et al., 2016).

Modern programming platforms, often originating from the computer game industry, make it relatively easy to model a virtual environment. Consequently, an increasing number of studies into robotic or neuromimetic control are carried out with virtual robots operating within a virtual environment. This approach is taken in the studies described in Edwards (2010). Dickson et al. (2006) describes an integrated model of Drosophila flight in which visual input is processed by an algorithmic (that is, non-neural) “brain” to control a biomechanically accurate representation of the fly. As the virtual fly traverses its environment, its visual input updates, closing the loop of the simulation. Cope et al. (2016b) is a recent study which omits the biomechanically accurate component of the model but could provide the biologically plausible brain to make a computational neurobehavioral fly model. Cofer et al. (2010a) use an environment called AnimatLab (Cofer et al., 2010b) to simulate the locust's jumping mechanism in an open-loop, software-only investigation. As this appears to be a feed-forward model without sensory feedback, we would describe it as an input-assumption model. N'Guyen et al. (2014) and Thurat et al. (2015) are two studies of the oculomotor system which model sensory input, neural control *and* motor output in software. These studies fall outside the remit of computational neurobehavior only because they omit to close the sensory loop. DeWolf et al. (2016) describes a reach model comprising a simplified virtual arm (with fewer degrees of freedom than a primate arm), and a biologically inspired brain model. This model also omits to close the sensory feedback loop and we consider it a computational neuroscience study of a (virtually) embodied model.

There are also experimental closed-loop approaches to understanding sensorimotor control. Ejaz et al. (2013) places a fly in a fixed position, and couples it with a free-to-move robot. The sensory input collected by the robot is projected onto the eyes of the fly, and activity from a selected neuron in the fly's brain is used to drive a control system for the robot's movements. This allows the experimenters to study the behavior of the fly's brain operating in a closed-loop condition that is more natural than the open-loop condition that many other experimental techniques mandate. The results from closed-loop experiments will undoubtedly inform future neurobehavioral models.

Thus, while there are many models that close the agent-environment loop and display partial biological plausibility, the biomimetic features are usually confined to a sub-system of the entire model. This leads us to formalize a definition of computational neurobehavior as: *The study of biological sensory-motor system behavior using biologically accurate models of sensory input, brain and motor sub-systems operating in a closed-loop*. We believe our oculomotor model is one of the first such models using this approach and shares many features with that of Arena et al. (2017) which describes a robotic insect system based on the fly species *Drosophila Melanogaster*. It has biomimetic insect legs implemented in a virtual robot and a neuromimetic brain. Closing the loop is a visual sensory input system which is able to determine the distance to an on-coming obstacle and the obstacle's height. The authors demonstrate that the virtual robot is able to learn to climb in a realistic manner and suggest it may be compared with experimental data from future Drosophila experiments addressing obstacle climbing and learning. The visual system is not described in detail, but if it is modeled in a biologically plausible manner, then this work may reasonably be described as a computational neurobehavior study, contemporaneous with our own.

## Author contributions

SJ, AB, and AC implemented existing parts of the model in SpineML. AB developed the saccade generator brainstem model. SJ performed the technical and scientific integration of the biomechanical eye. CP and KM developed the biomechanical eye model. SJ wrote the manuscript; SA, AB, KG, and KM contributed to the manuscript. KG conceived the project.

## Supplemental data

The model specification, results and all code required to reproduce the results of this work are available at: https://github.com/ABRG-Models/OMM_NeuroMuscular.

### Conflict of interest statement

The authors declare that the research was conducted in the absence of any commercial or financial relationships that could be construed as a potential conflict of interest.
